# A nature-inspired hydrogen-bonded supramolecular complex for selective copper ion removal from water

**DOI:** 10.1038/s41467-020-17757-6

**Published:** 2020-08-07

**Authors:** Ngoc T. Bui, Hyungmook Kang, Simon J. Teat, Gregory M. Su, Chih-Wen Pao, Yi-Sheng Liu, Edmond W. Zaia, Jinghua Guo, Jeng-Lung Chen, Katie R. Meihaus, Chaochao Dun, Tracy M. Mattox, Jeffrey R. Long, Peter Fiske, Robert Kostecki, Jeffrey J. Urban

**Affiliations:** 1 The Molecular Foundry, Lawrence Berkeley National Laboratory, Berkeley, CA 94720 USA; 2 Department of Mechanical Engineering, University of California, Berkeley, CA 94720 USA; 3 Advanced Light Sources, Lawrence Berkeley National Laboratory, Berkeley, CA 94720 USA; 4 National Synchrotron Radiation Research Center, Hsinchu Science Park, Hsinchu, 30076 Taiwan; 5 Departments of Chemistry, University of California, Berkeley, CA 94720 USA; 6 Materials Sciences Division, Lawrence Berkeley National Laboratory, Berkeley, CA 94720 USA; 7 Chemical and Biomolecular Engineering, University of California, Berkeley, CA 94720 USA; 8 Water-Energy Resilience Research Institute, Lawrence Berkeley National Laboratory, Berkeley, CA 94720 USA; 9 Energy Storage and Distributed Resources Division, Lawrence Berkeley National Laboratory, Berkeley, CA 94720 USA; 10 Present Address: The School of Chemical, Biological and Materials Engineering and the School of Civil Engineering and Environmental Science, the University of Oklahoma, Norman, OK 73019 USA

**Keywords:** Environmental impact, Coordination chemistry, Organic-inorganic nanostructures, Two-dimensional materials, Water resources

## Abstract

Herein, we present a scalable approach for the synthesis of a hydrogen-bonded organic–inorganic framework via coordination-driven supramolecular chemistry, for efficient remediation of trace heavy metal ions from water. In particular, using copper as our model ion of interest and inspired by nature’s use of histidine residues within the active sites of various copper binding proteins, we design a framework featuring pendant imidazole rings and copper-chelating salicylaldoxime, known as zinc imidazole salicylaldoxime supramolecule. This material is water-stable and exhibits unprecedented adsorption kinetics, up to 50 times faster than state-of-the-art materials for selective copper ion capture from water. Furthermore, selective copper removal is achieved using this material in a pH range that was proven ineffective with previously reported metal–organic frameworks. Molecular dynamics simulations show that this supramolecule can reversibly breathe water through lattice expansion and contraction, and that water is initially transported into the lattice through hopping between hydrogen-bond sites.

## Abstract (web summary)

Heavy metals and metalloids pose major threats to health and environmental ecosystems, thus systems for low-cost remediation are needed. Here the authors report the scalable design of a hydrogen-bonded organic–inorganic framework for selective removal of trace heavy metal ions from water.

## Introduction

Rapid population growth, industrialization, and climate variability are just a few of the factors that have contributed to pushing the global ecological balance near a threshold where immediate attention is required to ensure a future of sustainability. Addressing the inextricably linked water-energy-food security nexus is critical to promote societal stability and human prosperity^1^. Despite intensive efforts, access to safe drinking water in particular is still at risk, due in part to unacceptable levels of toxic heavy metals found in various water streams and food webs. Heavy metals and metalloids, released from geochemical cycles and anthropogenic activities, pose major threats to environmental ecosystems and can cause severe adverse health effects in humans, plants, and microorganisms. While some of these metals are essential for biological functions, they can also escape control mechanisms—such as transport, compartmentalization, and binding to designated cell constituents—via coordination chemistry and redox pathways, leading to misregulation of trafficking machinery and ion homeostasis^2,3^. Undesirable binding of heavy metals to DNA and nuclear proteins often leads to cell malfunction, oxidative deterioration of biological macromolecules, and eventually toxicity^3^. Effective solutions for efficient remediation of trace heavy metal ions from water with low costs are key pieces of the puzzle to promote environmental and human well-being.

Copper, one of the most widely used heavy metals, is toxic to humans in large quantities, despite its essential role in our physiology^4^. A copper imbalance can impact heart function and lipid metabolism as well as causing inflammation and resistance to chemotherapeutic drugs^5^. The U.S. Environmental Protection Agency has established a maximum level of copper in drinking water at 1.3 mg L^−1^^6^. However, copper levels reported in drinking water in Europe, Canada, and the USA can range from ≤0.005 to >30 mg L^−1^^6^. Historically, there have been several outbreaks of copper poisoning from ingestion of drinks that were contaminated with copper salts^7^. Given that millions of tons of copper are produced annually worldwide and it is ubiquitous across industries^8^, maintaining low levels of copper contaminants in water streams is a present concern. Therefore, seeking highly efficient approaches for copper removal from water is important. Beyond human health, selective separations involving copper is a relevant area of research for which traditional metallurgical solutions are limited. For example, in electrolytic nickel refining, selective removal of copper from nickel electrolysis anolytes has long been a problem plaguing the global metallurgical industry, and requires more effective solutions^9^.

Challenges inherent to heavy metal ion remediation from water include incomplete ion removal, high costs, and the generation of toxic, indistinguishable ion mixtures in sludge waste streams^10^. Indeed, isolating and disposing of only toxic contaminants from a myriad of species coexisting in water streams—some of which are valuable catalyst materials—appears to be a costly bottleneck for the water industry. Several methods have been used to remove heavy metal ions from wastewater, including evaporation, chemical precipitation, coagulation-flocculation, photocatalysis, and membrane separation, each with advantages and disadvantages^10,11^. Adsorption has been shown to be an effective and economic approach for heavy metal ion removal, as it offers flexibility in operation, a low volume of harmful secondary products, reversibility, and is capable of producing high-quality effluent with low energy and maintenance costs^10^. The efficacy of adsorption methods is limited largely by the ability to design an adsorbent with the necessary physical or chemical functionality for the contaminant of choice.

A promising ion adsorbent should exhibit high adsorption kinetics, large adsorption capacities even at low contaminant levels, selectivity for the target ion, stability in the water environment, scalability, regenerability, and affordability. Several materials have been shown to be effective for copper ion adsorption, such as carbon-based adsorbents^12,13^, polymer-based adsorbents^14,15^, layered metal sulfides^16,17^, porous aromatic frameworks^4^, and metal–organic frameworks^18–20^. The thioether-functionalized porous aromatic framework PAF-1-SMe was shown to be highly selective for the detection of copper ions in biological and aqueous samples^4^, but it was exclusively investigated as a diagnostic tool for the detection of Wilson’s disease. ZIF-8 was also recently reported to exhibit an extremely high Cu(II) adsorption capacity (up to ~800 mg g^−1^), establishing it among the state-of-the-art adsorbents for copper ion capture^18^. In general, however, most materials investigated to date for copper ion capture often suffer from one or more drawbacks, such as low selectivity, low capacity, or limited function (typically only in low acidic conditions (pH > 3)), and their syntheses have not been designed for affordable copper removal at scale, often using costly organic solvents or expensive preparations^19,20^. The design of selective adsorbents for efficient removal of ions with fast kinetics and high adsorption capacities, using simple and versatile synthetic routes for economic production at scale, is thus critical.

Hydrogen-bonded organic–inorganic frameworks (HOIF) are an emerging class of adsorbents that have recently been demonstrated for selective separations due to their flexibility and the highly reversible nature of their guest uptake^21–25^. Such promising results motivated us to explore and exploit crystal frameworks built predominantly on the propagation of hydrogen-bonding networks ubiquitously found in sophisticated functions performed by nature^26^ for selective ion removal. Herein, we present, zinc imidazole salicylaldoxime supramolecule (ZIOS), a HOIF that is capable of selective copper capture without the need for postsynthetic modification and exhibits excellent separation performance relative to ZIF-8. We couple aqueous coordination chemistry and supramolecular assembly to achieve a simple and scalable synthesis of ZIOS, which is composed of trinuclear units of zinc(II), 2-methylimidazole, and salicylaldoxime. Our material exhibits unprecedentedly fast adsorption kinetics as well as high adsorption capacities and selective performance at low pH values, rendering it a promising candidate for the adsorptive removal of copper from acid mine drainage-polluted water^27^. In addition, ZIOS crystals are highly stable in water, exhibiting no changes in crystallinity or structure after exposure to water for 52 days. Interestingly, this stability results from the ability of the material to reversibly breathe water through lattice expansion and contraction. We envision that such supramolecular structures may inform the further development of functional crystalline structures that can be translated to macroscopic platforms for selective removal of toxic metals from water.

## Results

### Design, synthesis, characterization, and adsorption tests

**Fig. 1:**
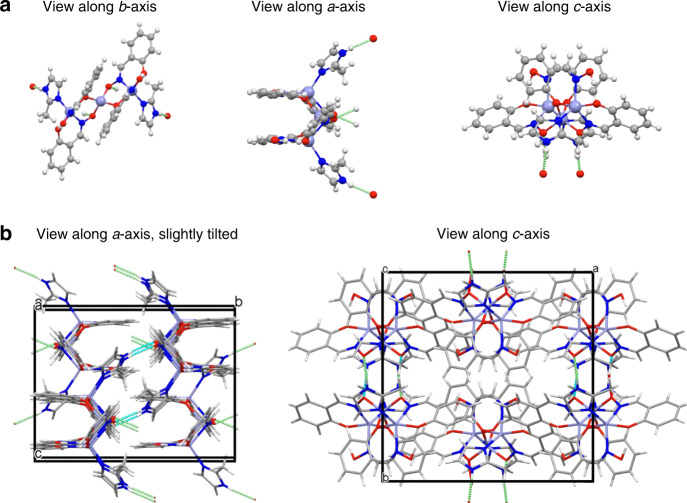
Structures of ZIOS. **a** Structures of a trinuclear unit in ZIOS along each crystallographic direction, as obtained from synchrotron X-ray diffraction data; lavender, red, blue, gray, and white spheres represent Zn, O, N, C, and H atoms, respectively. **b** Different views of the three-dimensional supramolecular structure of one ZIOS unit cell with observed hydrogen-bonding interactions (green/cyan dotted lines); a wireframe model is used for simplicity and clarity. In the solid state, nanochannels created between stacked layers of trimers are separated by ~2.7 Å, close to the typical length of a solid-state hydrogen bond (2–3 Å). Further details of the ZIOS structure are shown in Supplementary Fig. 1 and Supplementary Tables 1–3.

**Fig. 2:**
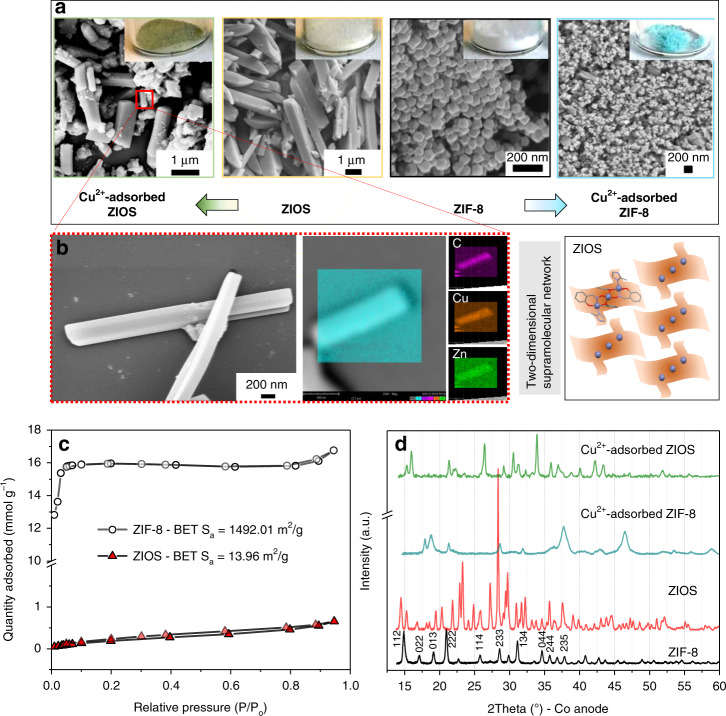
Structural, chemical, and physical properties of ZIOS and ZIF-8 before and after adsorption. **a** SEM images and optical properties of ZIOS and ZIF-8 before and after Cu^2+^ adsorption. **b** An expanded SEM image and energy-dispersive X-ray spectroscopy (EDS) analyses with area-scan on ZIOS-Cu (note that the samples were loaded on a silicon wafer, which is a source of Si, O, and N); and a schematic diagram of a ZIOS network created by periodic stacking of two-dimensional trinuclear units. **c** N_2_ adsorption and desorption isotherms and Brunauer–Emmett–Teller (BET) specific surface areas (S_a_) of ZIOS and ZIF-8. **d** PXRD data for ZIOS and ZIF-8 before and after 24-h exposure to aqueous Cu^2+^ (425 ppm solution) at ambient conditions. ZIOS clearly retains a high degree of crystallinity following copper adsorption, in contrast to ZIF-8.

We sought to design a HOIF through a new, simple, and scalable synthetic approach. In particular, the reaction of Zn(NO_3_)_2_, 2-methylimidazole (Hmim), and the copper chelator salicylaldoxime (H_2_salox) in water around 50 °C results in the formation of a zinc imidazole salicylaldoxime supramolecule, hereafter referred to as ZIOS. The material forms rapidly as small crystals in relatively high yield (76%) following the combination of separate mixtures of H_2_salox/Zn(NO_3_)_3_ and H_2_salox/mim. Synchrotron single-crystal X-ray diffraction (SC-XRD) data (Fig. 1a) revealed that the ZIOS structure features Zn_3_(Hsalox)_4_(mim)_2_ (i.e., Zn_3_(C_6_H_4_CHNOHO)_4_(CH_3_C_3_H_2_N_2_)_2_) trinuclear units, wherein H_2_salox and 2-methylimidazole are deprotonated and bonded directly to Zn^2+^ nodes through tetrahedral and pentahedral coordination by oxygen and nitrogen, respectively. Intermolecular hydrogen bonds between the hydroxyimino oxygen of H_2_salox and the pyrrolic nitrogen of 2-methylimidazole lead to the formation of a two-dimensional supramolecular network. The ZIOS structure is essentially nonporous, in contrast to MOFs and ZIFs^28^, with a Brunauer–Emmett–Teller (BET) surface area of ~12–14 m^2^ g^−1^ confirmed by both BET measurements at different activation conditions, e.g. 100 and 200 °C and grand canonical Monte Carlo simulations (Fig. 2c and Supplementary Fig. 2; c.f. BET surface area ~1200–1492 m^2^ g^−1^ for ZIF-8 determined in this work, see the Supporting Information). Even still, we find that the Cu^2+^ uptake capacity of ZIOS is 40% higher than that of a sample of ZIF-8 prepared in our hands, and the supramolecular structure exhibits unprecedented rapid adsorption kinetics (via infra). This surprising performance suggests the role of a chemisorption-based uptake mechanism, based on the coordination of Cu^2+^ by the aldoxime and histidine-inspired imidazole pendant groups present in the host structure. Powder X-ray diffraction characterization of ZIOS revealed that the bulk, as-synthesized material is highly crystalline (Fig. 2d). Thermogravimetric analysis data (Supplementary Fig. 6) collected under a N_2_ atmosphere revealed minimal (<5%) weight loss at ~50 °C, likely due to traces of uncoordinated water, followed by a two-step decomposition beginning at 250 °C, as a result of ligand degradation. Differential scanning calorimetry (DSC) analysis of ZIOS (Supplementary Fig. 7) further revealed exothermic peaks between ~220 and 250 °C corresponding to salicylaldoxime degradation.

Motivated by how nature performs complex functions based on the propagation of hydrogen-bonding networks, we examined whether ZIOS could function effectively in the capture of copper ions from water. Adsorption tests were performed on as-synthesized samples of ZIOS and ZIF-8 (see the Supporting Information) to observe their adsorption kinetics, isotherms, distribution coefficient, and selective performance in complex aqueous environments containing copper in the presence of several competing ions. Details of these experiments are listed in the Supporting Information. Samples of ZIOS and ZIF-8 were initially exposed to an aqueous CuCl_2_ solution (895 ppm, i.e., 425 ppm Cu^2+^, selected to benchmark the adsorptive performance of our adsorbents against ZIF-8 reported by Zhang et al.^18^) at the room temperature, and within the first 30 min both samples underwent a color change, from white to pale blue or green, respectively (Fig. 2a). This change is indicative of different interactions between Cu^2+^ and two frameworks, despite the presence of imidazole ligands in both structures. Scanning electron microscopy (SEM) images of ZIOS revealed that the material partially retains its rod-like structure following 24 h of exposure to Cu^2+^ (Fig. 2a, b), and energy-dispersive X-ray spectroscopy characterization revealed an even distribution of ions across the rod (Fig. 2b). In other domains of ZIOS-Cu, we observed a two-dimensional sheet-like structure (Supplementary Fig. 3) which is likely due to the sequence of sonication, vortex, copper adsorption, centrifugation, washing, and drying that the material underwent. The single-crystal structure of ZIOS-Cu was thus not obtained due to the lack of sufficiently large crystals that satisfy the minimal size requirement of the single-crystal X-ray diffraction technique. In contrast, after 24 h the ZIF-8 particles decrease in size and in general take on a different morphology (Fig. 2a). Crystal-to-crystal transformation due to postsynthetic treatments were previously reported for ZIF-8^29^. Powder X-ray diffraction characterization revealed that both materials undergo a transition to a new crystalline state upon Cu^2+^ uptake (Fig. 2c).

**Fig. 3:**
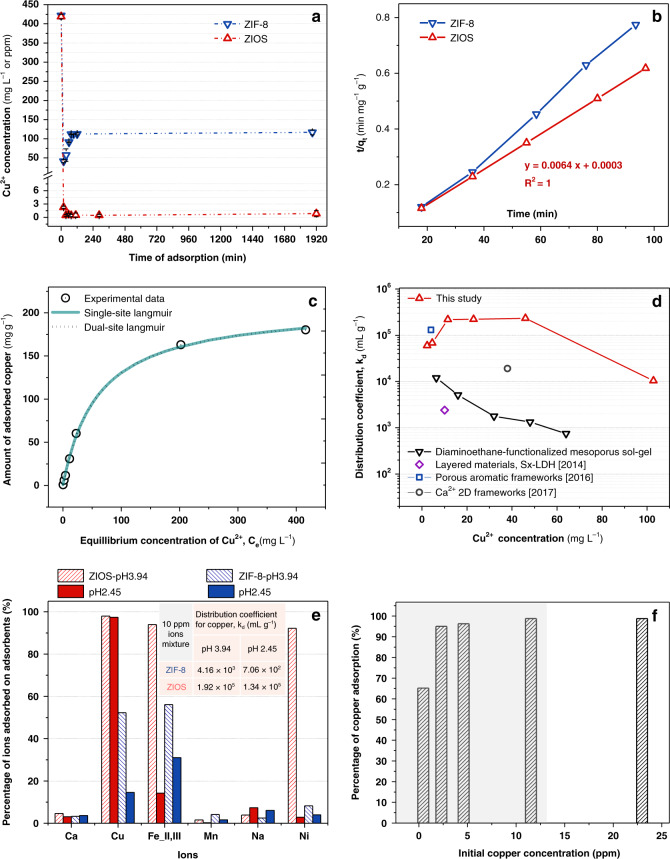
Adsorption behaviors of ZIOS and ZIF-8. **a**, **b** Copper (II) ion adsorption kinetics collected on ZIF-8 and ZIOS (error bars were obtained from three different adsorption tests conducted on materials synthesized from random batches). **c** Experimental copper adsorption isotherm for ZIOS (symbols) shown with fit using single-site (blue line) and dual-site (dotted line) Langmuir models. **d** Plots of distribution coefficients for ZIOS versus copper ion concentration (red triangles), compared with coefficients for other state-of-the-art copper ion adsorbents: a diaminoethane-functionalized mesoporous sol-gel (black triangles, ref. ^12^), double hydroxides intercalated with polysulfides (purple diamond, ref. ^32^), PAF-1-SMe (blue square, ref. ^5^), and a Ca^2+^ two-dimensional MOF (gray circle, ref. ^71^). **e** Copper selectivity of ZIOS and ZIF-8 when exposed to aqueous solutions (pH = 2.45 and 3.94) simultaneously containing equimolar concentration of Ca^2+^, Cu^2+^, Fe^2+^/Fe^3+^, Mn^2+^, Na^+^, and Ni^2+^ (present as Cl^−^ or NO_3_^−^ salts). **f** Copper(II) adsorption data for ZIOS at low copper concentrations. Uptake of copper(II) in ZIOS at these trace levels is excellent: ~65% at 0.45 ppm Cu^2+^ and ~95% at 2.5 ppm Cu^2+^.

Adsorption kinetics data for ZIF-8 and ZIOS in the presence of 895 ppm CuCl_2_ solution are shown in Fig. 3. In the case of ZIF-8 exposure, the copper concentration drops to ~42 ppm within the first 20 min before increasing slightly and reaching a steady state value of ~115 ppm after 75 min (Fig. 3a). Thus, although ZIF-8 initially adsorbs more than 80% of the copper ions present in solution, some of this copper is then gradually released (Fig. 3a). This behavior and the nonlinear pseudo-second-order adsorption kinetics (Fig. 3b) are indicative of weak interactions between copper(II) and ZIF-8. In contrast, addition of ZIOS to a ~425 ppm Cu^2+^ solution results in a significant drop in ion concentration to a steady state concentration <1.5 ppm in ~30 min (Fig. 3a), close to the 1.3 ppm upper limit for copper ions in drinking water. Further analyses revealed that copper capture by ZIOS occurs with exceptionally fast kinetics, with a pseudo-second-order adsorption rate constant of 155.3 ± 26.5 mg mg^−1^ min^−1^ (Fig. 3b). This rate is 50 times faster than that determined by us for ZIF-8 under the same conditions (3.4 ± 1.6 mg mg^−1^ min^−1^) and is significantly larger than rates reported for other state-of-the-art copper capture adsorbents (Supplementary Table 4). The positive linear dependence of the (t/q_t_) as a function of time shown in Fig. 3b is characteristic of pseudo-second-order adsorption of copper ions in ZIOS, confirming a chemisorption-governed process^30^. Notably, the fast adsorption kinetics exhibited by ZIOS are accompanied by a substantial adsorption capacity: at equilibrium, the amount of adsorbed copper ions (q_e_) was found to be 162.9 ± 9.4 mg g^−1^. For ZIF-8, a smaller equilibrium adsorption capacity of 116.4 ± 5.7 mg g^−1^ was determined from our measurements (material surface area = 1492 m^2^ g^−1^). We note this capacity for ZIF-8 is much lower than the uptake of ~800 mg g^−1^ reported previously (reported surface area = 1340 m^2^ g^−1^)^18^, although the authors in ref. ^18^ determined the material uptake after only 30 min of exposure to a copper ion solution, and our data indicate that equilibrium does not occur until ~75 min. As a comparison at this 30 min mark, the uptake of our sample of ZIF-8 was 151.7 ± 4.3 mg g^−1^. It is worth to note that the ratio of mass concentration (in mg L^−1^) of Cu: ZIF-8 used in our study is ~0.17:1, whereas that reported in Zhang and coworkers’ work is 1.26:1. We also note that adsorption capacity can be sensitive to slight differences in material preparation and activation conditions.

We further equilibrated ~2.5 mg ZIOS with solutions featuring a wide range of copper concentrations (~0.45 to ~420 ppm, equilibration time of 24 h). Each solution was then analyzed by inductively coupled plasma-optical emission spectrometry (ICP-OES) to determine the quantity of copper adsorbed, and the resulting data are presented in the adsorption isotherm in Fig. 3c. The data could be fit using both single- and dual-site Langmuir models, and the corresponding fitting parameters and correlation coefficients are tabulated in Supplementary Table 5. Both fits indicate the presence of a strong adsorption site with a moderate saturation capacity *q*_sat_ of ~208 mg g^−1^ (Supplementary Table 5), which is eight times higher than that of a commercially available thiol-functionalized resin, Duolite GT73 (BET surface area of 47.3 m^2^ g^−1^ and capacity of 25 mg g^−1^ at an equilibrium concentration of 160 ppm, see Supplementary Table 4)^31^. While this capacity for ZIOS is less than that of PAF-1-SMe (*q*_sat_ = 662 mg g^−1^)^4^, our material is advantageous in that it can be prepared through a much simpler synthetic route. In addition, copper uptake kinetics in ZIOS are ~30 times faster than in PAF-1-SMe^4^. Using the Langmuir constant, *K*_L_, obtained from single-site Langmuir model^16^, and Blanchard’s^32^ original proposed equation (see the Supporting Information), we determined that the Gibbs free energy of copper ion adsorption by ZIOS is −27.23 kJ mol^−1^, indicating that uptake is also thermodynamically favorable.

We investigated the copper ion selectivity of ZIOS and our ZIF-8 sample by evaluating ion uptake from synthetic water samples containing a mixture of Cu^2+^, Na^+^, Ca^2+^, Fe^2+^, Fe^3+^, Mn^2+^, and Ni^2+^ ions, prepared from chloride and nitrate salts of these cations (10 ppm concentration for each ion, solution pH of 3.94). Notably, even in the presence of these other ions, ZIOS adsorbed ~98.0 % of the copper(II) in solution, nearly double the amount adsorbed by ZIF-8 (52.3%), and uptake of Na^+^, Ca^2+^, Mn^2+^ was less than 10% in ZIOS and ZIF-8. However, we found that ZIOS also adsorbs substantial Fe^2+^/Fe^3+^ and Ni^2+^, namely ~93.9% and ~92.2%, respectively, compared with only ~56.1%, and ~8.3% for ZIF-8, respectively (Fig. 3e). This behavior is perhaps unsurprising given that salicylaldoxime is an excellent chelator for transition metal ions^33^. Although these results indicate that ZIOS will exhibit low copper ion selectivity in solutions that also contain iron and nickel ions, they also indicate that ZIOS may have potential for applications that target more than one toxic cation and/or the selective removal of other metal ions from select mixtures. If the limited selectivity of ZIOS is indeed governed by the salicylaldoxime ligand, this behavior should be tunable by changing the solution pH to a range favorable for more selective copper uptake by this chelator (pH < 3)^33^. Indeed, when adsorption tests were carried out using metal ion solutions prepared with pH = 2.45, copper uptake by ZIOS remained high (~97.5%) while the material adsorbed negligible iron (~14.3%) and nickel (~2.9%); in contrast, ZIF-8 adsorbed more iron (~30.0%) than copper (~14.6%) (Fig. 3e). Significantly, these results for ZIOS at lower pH may be promising for applications such as electrolytic nickel refining, where the separation of copper and nickel ions remains a substantial challenge^9^. For example, the Cu^2+^/Ni^2+^ separation factor achieved with ZIOS at pH 2.45 is 1281.1, while separation factors of 1.2 (pH = ~5) and 4.1 (pH = 4) were reported for the commercial resin Purolite S984^28^ and an *N*-octyl-2-aminomethylpyridine-functionalized chelating resin^34^ under equimolar concentrations of Ni^2+^ and Cu^2+^. We further evaluated the copper selectivity of ZIOS in an ion mixture having Zn^2+^ as one of the contaminants and found this behavior almost unchanged (Supplementary Table 6).

To further investigate the ion selectivity of ZIOS, we calculated the copper distribution coefficient (*K*_d_, mL g^−1^), or the ratio of copper in the adsorbed versus the solution phase (Fig. 3d), which represents the extent of adsorbent effectiveness in sequestering a target species and the selectivity of the adsorbent for that species^35^. A *K*_d_ > 5000 is considered very good^35^, ≥10^4^ mL g^−1^ is considered excellent^16^ and >5 × 10^4^ is considered outstanding^4,35^. Notably, ZIOS exhibits *K*_d_ values as high as 2.3 × 10^5^ across various Cu^2+^ concentration (Fig. 3d), and the distribution coefficient remains high in the presence of several competing ions and at different pH values (e.g., ~1.34 × 10^5^ at pH 2.45, see Fig. 3e). This performance either matches or exceeds that of state-of-the-art Cu^2+^ adsorbents^4,16,17,35^.

### Adsorption mechanism via spectroscopic characterization

**Fig. 4:**
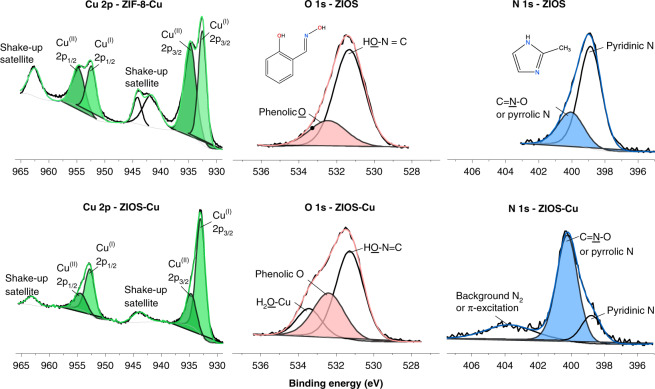
X-ray photoelectron spectroscopy results. XPS spectra for ZIOS before and after copper adsorption (Cu 2*p*, O 1*s*, and N 1*s* binding energy curves) and for ZIF-8 after copper adsorption (Cu 2*p* binding energies).

In order to better understand the mechanisms leading to copper ion uptake in ZIOS and ZIF-8, we utilized X-ray photoelectron spectroscopy (XPS), synchrotron radiation near-edge X-ray absorption fine structure (NEXAFS) spectroscopy, and extended X-ray absorption fine structure (EXAFS) spectroscopy to characterize the materials before and after copper ion adsorption. XPS analysis of ZIOS-Cu and ZIF-8-Cu was carried out in the binding energy regions of the Cu 2*p*, Zn 2*p*, N 1*s*, C 1*s*, and O 1s orbitals. In-depth analysis is shown in the Supporting Information. We note that for both materials, we observe signatures of copper(I) in addition to copper(II) (see below). All peak positions were referenced to the C 1*s* peak of adventitious carbon at 284.5 eV. Initial deconvolution of the Cu 2*p* spectra for both materials (Fig. 4) revealed similarities with spectra of a previously reported mixed-valence Cu^+^Cu^2+^ complex (for ZIF-8-Cu) and a Cu^+^ complex and/or a heteronuclear Cu^2+^Zn^2+^ complex (for ZIOS-Cu), both supported by macrocyclic ligands^36^. For ZIF-8-Cu, pairs of Cu 2*p*_1/2,3/2_ doublets (at 952.4/932.6 eV and 954.8/934.8 eV) and the charge transfer shake-up satellites (at 962.8 and 941.8–944.8 eV) were observed, indicative of Cu^+^ and Cu^2+^, respectively. For ZIOS-Cu, the Cu 2*p* spectrum exhibits peaks at 952.78 and 932.98 eV characteristic of Cu^+^ (2*p*_1/2_ and 2*p*_3/2_, respectively), in addition to weaker features at 954.48 and 934.78 eV that can be ascribed to Cu^2+^ (2*p*_1/2_ and 2*p*_3/2_, respectively). The copper(I) peaks could indicate the presence of a five-coordinate Cu^+^ diamagnetic adduct or a four-coordinate Cu^+^ complex with nitrogen macrocyclic ligands, while the copper(II) peaks are indicative of a heteronuclear Cu^2+^Zn^2+^ complex^37^.

In ZIOS, it is possible that copper chelates with salicylaldoxime by partially transmetalating with zinc nodes originally present in ZIOS and/or with 2-methylimidazole by coordinating with the free pyrrolic nitrogen. As seen in Fig. 4 and Supplementary Fig. 8, chelation of salicylaldoxime to Zn^2+^ in ZIOS results in a downshifting of the O 1*s* binding energies of free salicylaldoxime from 531.68 and 533.08 eV to 531.28 and 532.48 eV (HO–N and HO–C (phenol), respectively). Similar values of 531.28 and 532.38 eV (HO–N and HO–C (phenol), respectively) are observed following copper adsorption in ZIOS. In general, coordination to a metal ion results in a shifting of binding energies to lower values as a result of the electron donating/accepting ability of the metal ions^38^. For ZIOS, the 0.1 eV-downshift of the binding energy of the phenolic oxygen O 1*s* peak, along with the enhanced intensity of this peak after copper adsorption, show that this site plays an active role in scavenging copper ions. Moreover, the N 1*s* binding energy of ZIOS exhibits a downshift from 398.88 to 398.78 eV at the pyridinic nitrogen following copper adsorption and, interestingly, a significant increase in intensity of the peak at ~400.18 eV for C=N(–O) and/or a pyrrolic nitrogen (Fig. 4 and Supplementary Fig. 8). Often, fluctuations of peak intensity at a given chemical state are related to changes in the number of atoms in that state. The remarkable increase of the intensity ratio of I_(C=N)_/I_(C–N)_ from ~0.65 for ZIOS to ~1.54 for ZIOS-Cu indicates significant translational/rotational movement of C=N(–O) and/or pyrrolic nitrogen bonding from the bulk to the surface sites at which copper ions are coordinated. It remains unclear what imidazole nitrogen participates in copper coordination. Given the tautomeric property of the ionizable imidazole ring, both are possible if the Zn-imidazole bond was cleaved at the pyridinic nitrogen by copper ions. Note that the pH value of the stock copper chloride solution is ~5.5.

The roles of oxime and imidazole in coordinating with copper ions in ZIOS were further elucidated with NEXAFS spectroscopy. At the N K-edge, both ZIOS and ZIOS-Cu exhibit a sharp peak around 399 eV corresponding to a 1*s*→1π* transition. Both features are lower in energy than the corresponding 1*s*→1π* transition of the pyridinic nitrogen in the imidazole ring of the ZIF-8 structure (~400.5 eV). As the imino group typically shows a transition at 398–399 eV^39,40^, the peak at 399 eV for ZIOS and ZIOS-Cu likely stems from the N 1*s*→1π* transition of the oximate nitrogen (C=N–O) or the imidazolate pyridinic nitrogen^41^. After copper(II) ion adsorption in ZIOS, this peak for the 1*s*→1π* transition intensifies significantly compared with the broad peaks at 403.2, 406.3, and 410.5 eV, which correspond to the ionization of aromatic N–H and transitions from N 1*s*→σ*_N–H_ and σ*_C–N_, respectively^42^. This intensity increase suggests that the oxime hydroxyimino nitrogen and/or the imidazolate pyridinic nitrogen play a critical role in interacting with copper ions during adsorption. Given the close atomic radii of Zn (134 pm) and Cu (128 pm), along with the higher stability of complexes formed by copper(II) than zinc(II)^43^, there could be a transmetalation event where Cu^2+^ partially ion-exchanges with some of the Zn^2+^ nodes in ZIOS. During this event, the zinc–oxime bonds may be partially cleaved, leaving oxime ligands poised to scavenge copper ions. On the other hand, the unbound Lewis basic pyrrolic nitrogen atoms of the imidazole side chains in ZIOS should have a high affinity for Lewis acidic Cu^2+^ ions. These sites could actively coordinate with copper to form a CuZn-based heteropolynuclear complex, reactivity that could potentially mimic that found in Cu–Zn superoxide dismutase, an antioxidant enzyme found in almost all cells^26^ of the human body.

**Fig. 5:**
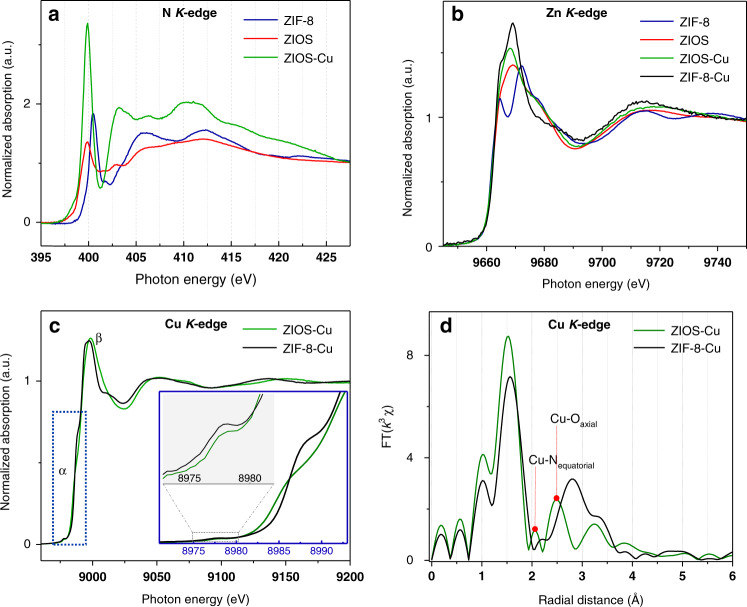
Near-edge X-ray absorption fine structure (NEXAFS) spectroscopy results. NEXAFS profiles near the **a** N K-edge, **b** Zn K-edge, and **c** Cu K-edge of ZIF-8 and ZIOS before and after copper adsorption. **d** Fourier transform of EXAFS data obtained for ZIOS-Cu and ZIF-8-Cu.

Zn K-edge NEXAFS profiles (Fig. 5b) and Fourier transformed spectra (Supplementary Fig. 9B) reveal that Cu^2+^ adsorption does not significantly change the local environment around the Zn^2+^ node in the ZIOS structure. This result is indicative of negligible adsorption of Cu^2+^ at sites already occupied by Zn^2+^ and preferential coordination at oxime and/or imidazole ligands. In contrast, a stark difference in the Zn K-edge data for ZIF-8 and ZIF-8-Cu (Supplementary Fig. 9B) indicates that the local environment around the Zn^2+^ nodes in ZIF-8 is altered upon Cu^2+^ adsorption, likely as a result of the observed structural transformation and consequent loss of characteristic ZIF-8 Zn^2+^ sites, observed via PXRD (Fig. 1d). We note that our results are in contrast with earlier reports on copper adsorption in ZIF-8^18^ and simple metal exchange reactions in zeolitic imidazolate frameworks^44^. Indeed, rather than maintaining the ZIF structure as a result of simple copper physisorption or metal exchange, all our data suggest the ZIF-8 sample is altered completely upon exposure to copper ions.

At the Cu K-edge (Fig. 5c), both ZIF-8-Cu and ZIOS-Cu show similar pre- and main-edge peaks at ~8978 eV (feature α, corresponding to a dipole-forbidden 1*s*→3*d* transition) and ~8985 and 8987 eV, respectively (feature β, predominantly governed by the dipolar transitions from 1*s*→4*p* empty states), which confirm the presence of Cu^2+^ in the samples^45,46^. However, a more detailed examination reveals some modest differences related to the peak intensities and sharpness between the two samples. The Cu L-edge NEXAFS profiles of the two samples (Supplementary Fig. 6C) also revealed differences in peaks positions. In NEXAFS, the energies and intensities of the peaks are typically sensitive to the local symmetry of metal ions, the metal coordination number, and oxidation state changes^45–47^. For example, ZIF-8-Cu exhibits a more intense feature α than ZIOS-Cu (Fig. 5c, inset) while the main-edge feature β is slightly less intense relative to that of ZIOS-Cu. This is indicative of 3*d*–4*p* mixing^45,48,49^, which is typically more pronounced in highly distorted, noncentrosymmetric structures. This property of ZIF-8-Cu is further supported by the broadness of peak β, which can be correlated with low coordination symmetry around copper^50^. Furthermore, the intense rising shoulder at ~8987 eV observed for ZIF-8-Cu, which is impacted by the ligand field symmetry, has been associated with the ligand–copper–ligand angle that is less than 180°^47,51^. Distinct patterns, except the first major peak at ~1.5 Å, resulting from Fourier transform of EXAFS data at the Cu K-edge further confirmed the existence of different copper coordination environments for ZIF-8-Cu and ZIOS-Cu (Fig. 5d). The first peak, observed in patterns of both samples, is associated with the copper first coordination shell comprising water oxygens, oxygen atoms of the nitrate anion and/or chloride ions^52,53^. However, it is unlikely that there are chloride ions in this coordination shell as at the diluted testing concentration of CuCl_2_ (895 ppm), CuCl_2_ is prone to fully dissociate in the aqueous solution, rendering the inner hydration shell of copper with water molecules and nitrate anions. ZIF-8-Cu shows another predominant peak at ~2.85 Å which is within range of reported Cu–C bonds^45^. Furthermore, there is no evidence of Cu–N bonding in ZIF-8-Cu, supporting the fact that the parent ZIF-8 structure featuring metal–imidazole bonding is completely transformed following copper uptake. On the other hand, peaks at ~2.01 and 2.41 Å for ZIOS-Cu (averaged from different scans) align well with reported Cu–N_equatorial_ and Cu–O_axial_ distances^45–48,54^, respectively, in a pentacoordinated complex with a square pyramidal geometry, suggesting some degree of chemisorption. Other peaks observed in the range of 3.2–3.9 Å for ZIOS-Cu correspond to Cu–C bonds^45,47^. From these analyses, we infer that copper predominantly chemisorbs in ZIOS by coordinating with the oxime and/or imidazole ligands, while copper ions in ZIF-8-Cu appear to be weakly bound through physisorptive interactions and also perhaps weakly coordinated within the structure. The extent to which oxime and imidazole moieties coordinate with copper in ZIOS-Cu remains undetermined.

From ICP-OES analysis of acid-digested solid samples, we were able to estimate that our samples of ZIF-8, ZIF-8-Cu, ZIOS, and ZIOS-Cu contain ~23.4, 19.9, 18.0, and 10.1 wt% Zn, respectively. Moreover, the ratios of Cu:Zn (by mass) in ZIF-8-Cu and ZIOS-Cu are 0.9:1 and 1.7:1, respectively. In these tests, ZIF-8-Cu and ZIOS-Cu were analyzed after soaking ZIF-8 and ZIOS for 24 h in a ~895 ppm CuCl_2_ solution at pH 5.5. These results indicate that a large amount of Zn^2+^ still remains in ZIF-8-Cu, in spite of the structural collapse observed via SEM and PXRD, indicating that ion exchange is not the main mechanism governing copper ion uptake in this material. Rather, our data indicate that copper adsorption in ZIF-8 leads to collapse of the ZIF-8 parent structure and the formation of a new phase that has retained the majority of the zinc ions as well as incorporated physisorbed and also possibly some chemically bound copper ions. In contrast, for ZIOS, a fraction of the Zn^2+^ ions appear to be replaced with copper ions, leading to a structure featuring Cu^2+^/Cu^+^ coordination pockets near the coordinated Zn^2+^ ions in ZIOS.

Although our data indicate that the salicylaldoxime and imidazole ligands in ZIOS contribute to its copper ion coordination capability, the precise mechanism of rapid copper scavenging remains unclear. We hypothesize that, upon immersion into a copper(II)-containing aqueous environment, the framework expands to some extent by gradually inviting water molecules to “assimilate” themselves into the framework through the propagation of a hydrogen-bonding network. This process may then open up channels that can accept copper ions. In addition to spontaneously coordinating with imidazole pendants at Lewis basic pyrrolic nitrogen sites, copper ions may partially exchange with the Zn^2+^ nodes of the trinuclear units, chelating with salicylaldoxime ligands and leading to a partial transformation of the crystalline structure. Adsorption evidently occurs quickly enough such that ZIOS does not have enough time to further interact with water and collapse. This behavior of ZIOS aligns well with the guest-binding property of metallohosts, structures that contain one or more transition metal atoms^55,56^. This property is regulated by metal coordination or redox reactions leading to site-selective transmetalation and ultimately to the construction of heteropolynuclear supramolecular structures. The transmetalation is evident by the amount of zinc ion transferred from ZIOS into the supernatant during copper adsorption quantified by ICP-OES (Supplementary Fig. 10).

### Behaviors and stability of ZIOS in aqueous environment

**Fig. 6:**
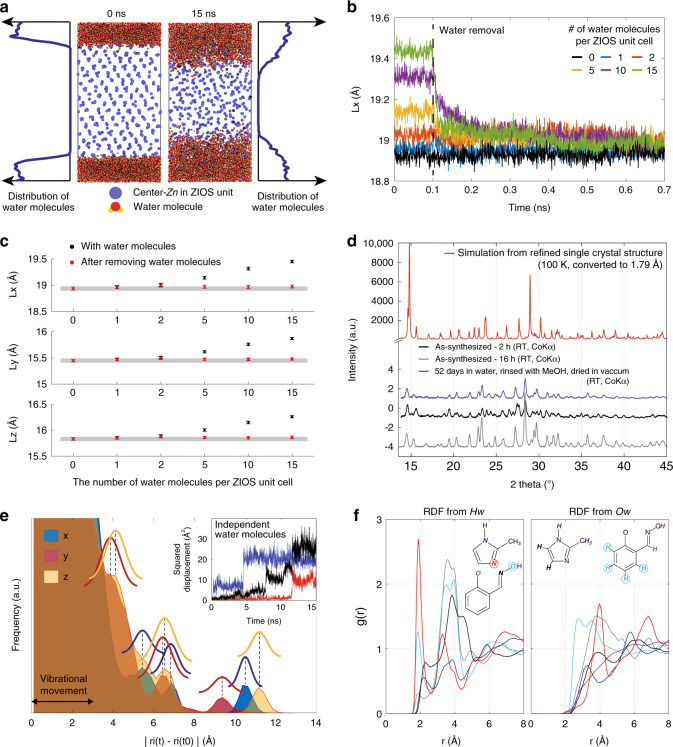
Behaviors of ZIOS in aqueous environment via molecular dynamics simulation and PXRD. **a** Water penetrates into ZIOS within nanoseconds, based on a flat two-dimensional channel simulation (4 × 4 × 8 ZIOS unit cells were originally placed with periodic 3-nm water channels). **b**, **c** For a periodic-boundary condition simulation box having 2 × 2 × 3 ZIOS unit cells and equilibrated state, simulations indicate clear lattice expansion when ZIOS is exposed to water and subsequent lattice contraction as water is removed. Simulations were performed separately with differing numbers of water molecules per ZIOS unit cell. **d** Comparison of a simulated ZIOS powder pattern from Rietveld refinement of synchrotron single-crystal X-ray diffraction data (red) with PXRD patterns of ZIOS obtained 2 h after synthesis (black), 16 h after synthesis (gray), and after being immersed in water for 52 days (blue). Notably, the powder pattern collected following extended water exposure is largely unchanged from the simulated pattern. **e** Water transport in ZIOS through hopping movement shown by histograms of spatial displacement of water molecules from their original positions (inset: squared displacement of independent water molecules with time). A representative case of ten water molecules per ZIOS unit cell is presented. **f** Radial distribution function showing the interaction of water molecules with the critical atoms in ZIOS unit via hydrogen bonds. The case of five water molecules per ZIOS unit cell was used in this analysis. The most active hydrogen-bond acceptor sites are highlighted.

To further support this hypothesis, we studied the behavior and stability of the highly crystalline ZIOS framework in an aqueous environment both computationally and experimentally. Molecular Dynamics (MD) simulations were performed using the LAMMPS package with standard three-dimensional periodic-boundary conditions. Further details are listed in the Experimental. Our results show that immersion of ZIOS unit cells in water leads to an expansion of the crystal lattice and the formation of nanochannels that can accommodate water molecules through the propagation of a hydrogen-bonding network. Water incorporation occurs quite rapidly—a snapshot at 15 ns (Fig. 6a) shows a noticeable amount of water molecules intercalating into ZIOS channels. Furthermore, as the number of water molecules introduced into the ZIOS unit cell increases, a gradual lattice expansion occurs in all crystallographic directions—L_x_, L_y_, and L_z_ (Fig. 6b, c). When water is removed, the lattice contracts back to its original size within less than 1 ns (Fig. 6b, c), indicating the expansion is reversible. Indeed, powder X-ray diffraction characterization of a sample of ZIOS soaked in water for 52 days, washed with methanol, and dried in vacuum revealed an almost identical pattern to that simulated from single-crystal X-ray diffraction data (Fig. 6d). A slight shift of the peaks is observed due to different diffraction scans temperatures (room temperature for the PXRD data versus 100 K for the SC-XRD data). This result serves as an experimental evidence of the breathing phenomena, suggested by the MD simulation, where ZIOS recovers its original crystalline structure after being excessively exposed to water for ~2 months. Other experimental approaches are however necessary to observe this truly interesting behavior in-operando. Still, to an extent, the breathing behavior of ZIOS is analogous to what was reported for some metal–organic frameworks that can adapt their pore opening to accommodate guest species, leading to dramatic change in cell volume without a loss of crystallinity or bond breaking^57,58^. In our cases, the new material does not just behave instantaneously. Instead, it held its structure through vigorous treatment steps. The impressive structural integrity of ZIOS supports the presence of a strikingly robust hydrogen-bonded network which helps the material to reversibly breathe through expansion–contraction cycles before and after exposure to water.

Interestingly, our simulation further shows that, when a few water molecules are first introduced into ZIOS, they move within the framework by hopping between various hydrogen-bonding sites, shown by discrete step changes in Fig. 6e and Supplementary Fig. 12. We define a channel in ZIOS as the simplest two-dimensional stack between two consecutive trinuclear subunits with an average size of 2.7 Å (calculated from our single-crystal X-ray diffraction data). These hopping events were observed for all our simulations, involving 1, 2, 5, 10, or 15 water molecules, especially in the initial time frames. When enough water molecules are introduced into the structure and there is enough time for sufficient lattice expansion, water begins to diffuse within the crystal structure. The transition from a hopping to a diffusion mechanism is best illustrated in the case of simulations involving 10 or 15 water molecules. As it is unclear when the onset of this transition to diffusion occurs, we report simulation data collected before and after equilibrium (Fig. 6e and Supplementary Fig. 12). Note that the step size of each hopping moment varies due to the spatiotemporal randomness of such events, which are dependent on the total average number of water molecules and diverse gaps between hydrogen-bonding sites. In ZIOS, the most active hydrogen acceptor site was determined to be the pyridinic imidazole nitrogen, based on the radial distribution function, *g*(r) (see Fig. 6f and the Supplementary Information). Meanwhile, the most active hydrogen donor sites are the hydrogen atoms of the hydroxyimino groups. From our simulations, the hydrogen-bonding peak was observed within 2 Å for the most active sites (Supplementary Fig. 13). Similar to previously reported^59,60^, we found evidence of the contribution of the benzene rings to ZIOS molecular associations through the hydrogen-bonding network. Although a water-benzene hydrogen bond is weaker than a water–water hydrogen bond, water molecules associated with benzene were believed to have more rotational and translational freedom than their water–water counterparts^60^. Further exploration is essential to confirm this hopping behavior experimentally.

## Discussion

In summary, by coupling coordination chemistry and supramolecular assembly, we have synthesized a HOIF, ZIOS, for the selective capture of copper ions from an aqueous environment. Inspired by how proteins capture copper ions through the imidazole side chains of polypeptidyl histidine, we designed a material that contains pendant imidazole rings that are poised to scavenge copper ions. The synthesis of this material is in principle highly scalable, and furthermore, as a result of structural copper-chelating salicylaldoxime ligands, ZIOS exhibits unprecedented rapid adsorption kinetics (30–50 times faster than those of current state-of-the-art copper adsorbents), and a moderately high copper ion adsorption capacity (eight times that of the commercial copper adsorbent Duolite GT73). Furthermore, selective copper removal is possible with ZIOS at pH <3, a value inaccessible with previously reported metal–organic frameworks for copper adsorption. Unlike a majority of metal–organic frameworks, our hydrogen-bonded framework is also stable to water exposure for several weeks. MD simulations show that this framework reversibly breathes in the presence of water through lattice expansion and contraction. Increasing the concentration of water in the unit cell further results in a transition from hopping to diffusion-based transport. Future efforts will investigate the regenerability of our material through copper adsorption–desorption cycling. More broadly, this study is a valuable starting point to guide further development of novel organic–inorganic hybrids that can be effectively leveraged to address at-scale removal of toxic metals from water.

## Methods

### Materials and chemicals

Zinc(II) nitrate hexahydrate (≥98%), 2-methylimidazole (>99%), salicylaldoxime, copper(II) chloride dehydrate, calcium(II) nitrate tetrahydrate, iron(II) chloride tetrahydrate, iron(III) chloride, nickel(II) nitrate hexahydrate, manganese(II) chloride, and sodium chloride were purchased from Sigma-Aldrich and used as received. Anhydrous methanol (>99.8%, Sigma-Aldrich) and Milli-Q water (18.2 MΩ cm^−1^) were used in the synthesis of ZIF-8/ZIOS and ion solution preparations, respectively.

### ZIF-8 synthesis

ZIF-8 crystals were prepared following the procedure reported by Song and coworkers^61^. Briefly, Zn(NO_3_)_2_‧6H_2_O (0.438 g, 1.47 mmol) and 2-methylimidazole (0.972 g, 11.8 mmol) were separately dissolved in methanol (20 mL) and then mixed together under ambient conditions with vigorous stirring for 4 h. Solid ZIF-8 was then separated from the suspension by centrifuging for 5 min at 8000 rpm and removal of the reaction solvent. The resulting solid was then rinsed with methanol (20 mL), centrifuged, and again separated from the solvent. This rinsing process was repeated twice more, and the final product was dried in a vacuum oven at the room temperature for 18 h.

### ZIOS synthesis

ZIOS crystals were synthesized from an aqueous solution of Zn(NO_3_)_2_‧6H_2_O (0.222 g, 0.75 mmol), 2-methylimidazole (0.489 g, 5.95 mmol) and salicylaldoxime (0.623 g, 4.54 mmol) for 2 h at 53–57 °C and atmospheric pressure. ZIOS was then separated from the reaction suspension by centrifuging for 5 min at 8000 rpm, rinsed with deionized water (25 mL), centrifuged, and reisolated; this rinsing was repeated two times. The resulting solid was finally rinsed once with methanol (20 mL) and isolated via centrifuge under the same conditions. The final product was then isolated by decanting the methanol and dried in a vacuum oven at the room temperature for 18 h. Single-crystal X-ray diffraction confirmed that the structure of ZIOS contains Zn_3_(L_1_)_4_(L_2_)_2_ trimers, where L_1_ is salicylaldoxime and L_2_ is 2-methylimidazole. (Supplementary Tables 1–3) and XPS confirmed elemental composition (Supplementary Fig. 4).

### Structural, physical, and chemical characterization

Further details are reported in the supporting information. Briefly, after synthesized, ZIF-8 and ZIOS were grinded with a mortar and pestle before characterization and adsorption tests. The crystal structure of ZIOS was elucidated with synchrotron SC-XRD collected on beamline 12.2.1 at the Advanced Light Source using a Bruker D8 diffractometer with a PHOTONII CPAD detector, with λ = 0.7288 Å, equipped with an Oxford Cryosystems Cryostream 800 plus, at 100 K. Representative morphology of MOFs before and after Cu^2+^ adsorption was observed with Zeiss Gemini Ultra-55 analytical field emission scanning electron microscope. Thermal behaviors of the samples with respect to dehydration, oxidation, decomposition, phase changes, melting point, and/or other heat-related events were observed with thermal gravimetric analysis in argon atmosphere and DSC in N_2_ atmosphere at a ramping rate of 5 °C min^−1^. Powder X-ray diffraction was conducted at 35 kV/40 mA with a CoKα wavelength of 1.79 Å. Surface areas were determined using BET analysis of N_2_ adsorption isotherms obtained at 77 K (Tristar 3020) after degassing samples at 100 °C for 20 h. Chemical and electronic states of elemental compositions of MOFs before and after Cu^2+^ adsorption were evaluated with XPS collected with a monochromated Al Kα source. All peak positions were referenced to the C 1*s* peak at 284.5 eV. Synchrotron soft- and hard- X-ray absorption (with NEXAFS, EXAFS, and Fourier transformed data) were conducted to understand the local environment surrounding Zn and Cu metal nodes to provide insights into adsorption mechanisms. ICP-OES test was used to quantify the total trace amount of ions in the supernatant after a given time of adsorption.

### Adsorption capability of ZIOS

ZIF-8 (as a control material) and ZIOS were evaluated with different adsorption tests from which experimental data were fitted accordingly to extract adsorption performance and behavior of MOFs. In essence, adsorption kinetics test was conducted where results were fitted with a pseudo-second-order model to determine the rate of adsorption and the amount of copper ion adsorbed on MOFs at equilibrium. Adsorption isotherm was then obtained by challenging ZIOS with solutions having copper concentration values covered a wide range from 0.45 to 450 ppm for 24 h. Results were then fitted with single-site and dual-site Langmuir models. Results from this study was also used to determine the distribution (or partition) coefficient *k*_d_ as well as the Gibbs free energy of copper ion adsorption on ZIOS. Furthermore, these MOFs were challenged with a selectivity test in which solutions having several cations and anions and adjusted pH were used. Details of experiments and calculations are listed in the supporting information.

### Molecular dynamics (MD) simulation

All-atom AMBER force fields for potential energy *U* were used in the MD simulation of this system.

1 $${U_{potential}} 	={\,} \mathop {\sum}\limits_{i > j} {\left[ {4\varepsilon _{ij}\left\{ {\left( {\frac{{\sigma _{ij}}}{{r_{ij}}}} \right)^{12} - \left( {\frac{{\sigma _{ij}}}{{r_{ij}}}} \right)^6} \right\} + \frac{{q_iq_j}}{{4\pi \varepsilon _0\varepsilon _rr_{ij}}}} \right]} \\ 	{\,\,}\quad + \mathop {\sum}\limits_{bonds} {K_r(r - r_0)^2} + \mathop {\sum}\limits_{angles} {K_\theta (\theta - \theta _0)^2} \\ 	\quad {\,\,\,}{ + \mathop {\sum}\limits_{torsions} {\frac{{K_\varphi }}{2}\left\{ {1 + \cos (n\phi - \gamma )} \right\}} + \mathop {\sum}\limits_{impropers} {K_\chi (\chi - \chi _0)^2} }.$$

In Eq. (1), the first term describes the nonbonded interactions including Van der Waals (VdW) as the Lennard-Jones 12-6 form and Coulombic forces from atom-centered partial charges. The following terms in the potential energy equation represent, respectively, bonds, angles, and proper and improper torsional interactions. To investigate the supramolecular coordinating complex, the cationic dummy atom model was employed for the zinc cation with a tetrahedral structure^62,63^. The force field parameters of atomistic oxidized salicylaldoxime and methylimidazole were developed in previous work^64,65^ and summarized in Supplementary Tables 7–11. The SPC/E water model^66^ was employed for water molecules, which depicts well the dynamic and structural properties of liquid water. The VdW interaction parameters between unlike atoms were obtained by the Lorentz–Berthelot combining rule. The nonbonded interactions separated by exactly three consecutive bonds (1–4 interactions) were reduced by related scaling factors^67,68^, which were optimized as 0.50 for VdW interactions and 0.83 for electrostatic interactions, respectively. Electronegativity equalization method^69^ was used to calculate the atomic charges by using *Multiwfn*^70^. The schematic molecular structures and partial charges of the components for the ZIOS unit and SPC/E water molecule are presented in Supplementary Fig. 11. MD simulation was performed using LAMMPS package with standard 3D periodic-boundary conditions. The nonbonded interactions were cut off at 12 Å while the Particle Mesh Ewald summation method was applied to treat the long-range electrostatic interactions. All simulations were carried out at isothermal–isobaric conditions, at 300 K and ambient pressure, in the Nose-Hoover NPT ensemble with time coupling constants of 100 and 1000 fs, respectively. After initial relaxations with short time steps and an equilibration with long time steps, at least 15 ns simulation of the ensemble were performed with a fixed time step of 1.0 fs. The atomic trajectories of simulation or the RDFs were recorded with an interval of 2 ps for post analysis.

## Supplementary information

Supplementary Information

Peer Review File

## Data Availability

All data generated or analyzed during this study are included in this published article, its supplementary information files, and are available from the corresponding author on reasonable request.
