# Supplementary material for: A nature-inspired hydrogen-bonded supramolecular complex for selective copper ion removal from water
Source: Nat Commun. 2020 Aug 7;11:3947. doi: 10.1038/s41467-020-17757-6 (PMC7415137; doi:10.1038/s41467-020-17757-6)
Supplement: Supplementary file 1 — Supplementary Information [file 41467_2020_17757_MOESM1_ESM.pdf]

Supporting Information for:

A nature-inspired hydrogen-bonded supramolecular complex for selective copper ion removal from water

Ngoc T. Bui[1,10,§], Hyungmook Kang[1,2,§], Simon J. Teat[3], Gregory M. Su[3], Chih-Wen Pao[4], Yi-Sheng Liu[3], Eddy Zaia[1], Jinghua Guo[3], Jeng-Lung Chen[4], Katie R. Meihaus[5], Chaochao Dun[1], Tracy M. Mattox[1], Jeffrey R. Long[5,6,7], Peter Fiske[8], Robert Kostecki[9], Jeffrey J. Urban[1,*]

[1] *The Molecular Foundry, Lawrence Berkeley National Laboratory, Berkeley, CA 94720, USA*
[2] *Department of Mechanical Engineering, University of California, Berkeley, CA 94720, USA*
[3] *Advanced Light Sources, Lawrence Berkeley National Laboratory, Berkeley, CA 94720, USA*
[4] *National Synchrotron Radiation Research Center, Hsinchu Science Park, Hsinchu 30076, Taiwan*
[5] *Departments of Chemistry, University of California, Berkeley, CA 94720, USA*
[6] *Materials Sciences Division, Lawrence Berkeley National Laboratory, Berkeley, CA 94720, USA*
[7] *Chemical and Biomolecular Engineering, University of California, Berkeley, CA 94720, USA*
[8] *Water-Energy Resilience Research Institute, Lawrence Berkeley National Laboratory, Berkeley, CA 94720, USA*
[9] *Energy Storage and Distributed Resources Division, Lawrence Berkeley National Laboratory, Berkeley, CA 94720, USA*
[10]*Current address: The School of Chemical, Biological and Materials Engineering and the School of Civil Engineering and Environmental Science, the University of Oklahoma, Norman, OK 73019, USA*

**Corresponding author: jjurban@lbl.gov*

ORCID: Ngoc T. Bui (0000-0003-4810-9534), Hyungmook Kang (0000-0002-2313-2175), Simon J. Teat (0000-0001-9515-2602), Gregory M. Su (0000-0001-7495-8041), Jeffrey J. Urban (0000-0003-4909-2869)

Supplementary Methods:

1. Materials Characterization

Scanning electron microscopy (SEM)

Representative surface morphology of ZIOS and ZIF-8 before and after Cu^{2+} adsorption was imaged using a Zeiss Gemini Ultra-55 Analytical Field Emission Scanning Electron Microscope (FESEM). Samples were first sputter coated with a thin layer of gold before imaging to obtain better contrast and to avoid charge accumulation. Energy-dispersive X-ray spectroscopy (EDS) was conducted on a Phenom Tabletop SEM at 15 kV in mapping mode.

Thermal gravimetric analysis (TGA)

TGA was conducted on a TA Instrument Q5500 TGA-MS under an argon atmosphere, with a ramping rate of 5 ^{o}C min^{-1}, to observe thermal behaviors of the materials related to dehydration, oxidation, or decomposition.

Differential Scanning Calorimetry (DSC)

DSC analyses were conducted from 25–400°C under a N_2 atmosphere with a ramping rate of 5°C min^{-1}, to observe thermally-induced physical transitions of the materials due to phase changes, melting, etc.

X-ray photoelectron spectroscopy (XPS)

XPS spectra were collected using a Thermo Scientific K-Alpha XPS System with a monochromated Al Kα source. A pass energy of 100 eV was used for survey scans and 20 eV for high resolution scans, with an energy resolution of 0.1 eV. Thin film surfaces were cleaned using an argon cluster gun (6000 eV, 150 atoms per cluster) for 60 seconds before all XPS measurements. All peak positions were referenced to the C 1s peak of adventitious carbon at 284.5 eV. XPS analysis of ZIOS-Cu and ZIF-8-Cu was carried out in the binding energy regions of the Cu 2p, Zn 2p, N 1s, C 1s and O 1s orbitals. We note that for both materials, we observe signatures of copper(I) in addition to copper(II) (see below). The presence of copper(I) could result from autoreduction of initial copper(II) in solution or the solid state, or during the XPS data collection itself. Initial deconvolution of the Cu 2p spectra for both materials (Fig. 4) revealed similarities with spectra of a previously reported mixed-valence $Cu^{+}Cu^{2+}$ complex (for ZIF-8-Cu) and a Cu^{+} complex and/or a heteronuclear $Cu^{2+}Zn^{2+}$ complex (for ZIOS-Cu), both supported by macrocyclic ligands.[1] For ZIF-8-Cu, pairs of Cu $2p_{1/2,3/2}$ doublets were observed at 952.4 / 932.6 eV and 954.8 / 934.8 eV, indicative of Cu^{+} and Cu^{2+}, respectively. The presence of charge transfer shake-up satellites at 962.8 and 941.8–944.8 eV further indicates the presence of both Cu^{+} and Cu^{2+} in ZIF-8-Cu. Additionally, the nearly equal intensity of the Cu^{+} and Cu^{2+} peaks and the broader Cu^{2+} component peak compared to the Cu^{+} peak (due to the broadening induced by multiplet splitting effects[2]) further suggest the presence of a delocalized, mixed-valence Cu^{+} / Cu^{2+} complex structure that contains very weakly coupled metal centers.[1,3]

For ZIOS-Cu, the Cu 2p spectrum exhibits peaks at 952.78 and 932.98 eV characteristic of Cu^{+} ($2p_{1/2}$ and $2p_{3/2}$, respectively), in addition to weaker features at 954.48 and 934.78 eV that can be ascribed to Cu^{2+} ($2p_{1/2}$ and $2p_{3/2}$, respectively). The copper(I) peaks could indicate the presence of a five-coordinate Cu^{+} diamagnetic adduct or a four-coordinate Cu^{+} complex with nitrogen macrocyclic ligands, while the copper(II) peaks are indicative of a heteronuclear $Cu^{2+}Zn^{2+}$ complex.[3] The weak feature at 934.78 eV and the subtle occurrence of satellite structures at ~943.9 and 963.2 eV, compared to the more pronounced features for ZIF-8-Cu, suggest that ZIOS-Cu contains a smaller fraction of Cu^{2+} on its surface than ZIF-8-

Cu. It is possible that ZIOS-Cu features coordination pockets occupied by a single Cu^{+} and that a small amount of Cu^{2+} remains on the surface from initial adsorption. ZIOS-Cu may also feature $Cu^{2+}Zn^{2+}$ coordination pockets in its structure, a possibility reinforced by the Cu 2p spectrum and Zn $2p_{3/2}$, N 1s, and C 1s peaks in the corresponding spectra at 1022.1, 400.28, and 285.58 eV (Supplementary Fig. 8), respectively.[1] Additionally, the slow scan of the ZIOS-Cu surface with X-rays could induce one-electron reduction of such heteronuclear $Cu^{2+}Zn^{2+}$ complex to a diamagnetic d^{10}–d^{10} $Cu^{+}Zn^{2+}$ species,[1] which could also explain the predominant Cu^{+} 2p peaks at 932.98 and 952.78 eV. Such a reduced species would not show any satellite structure except when the $Cu^{2+}Zn^{2+}$ is still present in the sample.

In all, adsorbed copper ions in ZIOS-Cu appear to be present as Cu^{+}–nitrogen macrocycle complexes and/or $Cu^{2+}Zn^{2+}$ heteronuclear complexes. It is possible that copper chelates with salicylaldoxime by partially transmetalating with zinc nodes originally present in ZIOS and/or with 2-methylimidazole by coordinating with the free pyrrolic nitrogen. As seen in Fig. 4 and Supplementary Fig. 8, chelation of salicylaldoxime to Zn^{2+} in ZIOS results in a downshifting of the O 1s binding energies of free salicylaldoxime from 531.68 and 533.08 eV to 531.28 and 532.48 eV (H**O**–N and H**O**–C (phenol), respectively). Similar values of 531.28 and 532.38 eV (H**O**–N and H**O**–C (phenol), respectively) are observed following copper adsorption in ZIOS. In general, coordination to a metal ion results in a shifting of binding energies to lower values as a result of the electron donating/accepting ability of the metal ions.[4] For ZIOS, the 0.1 eV-downshift of the binding energy of the phenolic oxygen O 1s peak, along with the enhanced intensity of this peak after copper adsorption, show that this site plays an active role in scavenging copper ions. Moreover, the N 1s binding energy of ZIOS exhibits a downshift from 398.88 to 398.78 eV at the pyridinic nitrogen following copper adsorption and, interestingly, a significant increase in intensity of the peak at ~400.18 eV for C=**N**(–O) and/or a pyrrolic nitrogen (Fig. 4 and Supplementary Fig. 8). In contrast, the N 1s binding energies in ZIF-8 shift to higher energies after copper adsorption. Often, fluctuations of peak intensity at a given chemical state are related to changes in the number of atoms in that state. The remarkable increase of the intensity ratio of $I_{(C=N)}/I_{(C–N)}$ from ~0.65 for ZIOS to ~1.54 for ZIOS-Cu indicates significant translational/rotational movement of C=N(–O) and/or pyrrolic nitrogen bonding from the bulk to the surface sites at which copper ions are coordinated. This behavior was consistently observed for two distinct ZIOS-Cu samples, prepared from separate syntheses and adsorption batches. It remains unclear what imidazole nitrogen participates in copper coordination. Given the tautomeric property of the ionizable imidazole ring, both are possible if the Zn-imidazole bond was cleaved at the pyridinic nitrogen by copper ions. Note that the pH value of the stock copper chloride solution is ~ 5.5.

Inductively coupled plasma – optical emission spectrometry (ICP-OES)

Inductively Coupled Plasma – Optical Emission Spectrometry test, performed on Varian ICP-OES 720 Series, was used to quantify the total trace ions in the supernatant after a given time of adsorption. Samples were collected and diluted with 2 wt% HNO_3 before ICP tests. 1000 ppm copper standard solution (Sigma Aldrich) was used to prepare diluted standard solutions having copper concentrations ranging from 0.1 to 20 ppm.

N_2 adsorption isotherms and surface area measurements

N_2 adsorption data were collected on an automated three station Tristar 3020 analyzer, connected to a regulated high pressure N_2 tank. Powder samples were degassed in vacuum at 100 °C for 20 h before data collection. To validate the non-porous property of the newly reported material like ZIOS, we also

measured the BET surface area of ZIOS at another activation condition (e.g., 200 ^{o}C for 20 h). At this condition, interestingly, we obtained a surface area value that replicates almost exactly that measured for ZIOS after being activated at 100 oC (e.g. ~13.95 m^2 g^{-1}). This result reinforced the non-porous property of ZIOS.

Furthermore, we theoretically calculated the accessible surface area of ZIOS. Walton and Snurr[5] proposed and proved that the BET surface areas calculated from the simulated adsorption isotherm (with grand canonical Monte Carlo – GCMC BET) agree very well with the accessible surface areas calculated directly from the crystal structures in a geometric fashion. Specifically, the accessible surface areas were calculated from a simple numerical integration technique where a probe molecule is "rolled" over the framework surface. The fraction of the probe molecules that did not overlap with other framework atoms was then used to calculate the accessible surface area (J. Phys. Chem. C., 2007, 111, 15350-15356). The diameters of the framework atoms for ZIOS were taken from the value of Lennard-Jones parameter, σ, which were used for our MD simulations. As a reference, the value of ZIF-8 was also calculated with the forcefield from (J. Phys. Chem. C, 2011, 116, 933-938). All the accessible surface area below was calculated with a probe diameter of 3.681 Å for nitrogen. The snapshots of framework for the coordination information were collected from the MD simulation.

The accessible surface area of:

ZIOS is 12.36 ±1.66 m^2 g^{-1} (20 snapshots' average)

ZIOS after expansion and contraction is 11.07 ±1.66 m^2/g (20 snapshots' average)

ZIF-8 is 1193 ±7 m^2 g^{-1} (50 snapshots' average)

As shown, the values achieved from our theoretical calculation closely represent the experimentally measured surface area (e.g. 13.95 m^2 g^{-1} for ZIOS and for 1492 m^2 g^{-1} ZIF-8). In sum, these results confirm the non-porous properties of our newly designed material – ZIOS.

Powder X-Ray Diffraction (XRD)

Powder XRD analyses were conducted on a Bruker AXS D8 Discover GADDS X-Ray Diffractometer equipped with a Vantec-500 area detector and operated at 35kV/40mA with a CoKα wavelength of 1.79 Å. Powder samples were loaded onto a silicon substrate for this test.

Near Edge Soft X-ray Absorption (Soft X-ray NEXAFs)

Substrates for transmission near edge X-ray absorption fine structure (NEXAFS) were prepared by coating silicon nitride windows with a thin layer of polystyrene. Silicon nitride windows (2 × 2 mm and 150 nm thick in a silicon supporting frame) were spin-coated with a 5 mg mL^{-1} solution of polystyrene in toluene at a spinning rate of 2,000 rpm for 45 s. This polystyrene layer was used to improve the adhesion between ZIOS or ZIF-8 and the substrate under vacuum. A well-dispersed suspension of adsorbent in hexane was then drop-cast onto the polystyrene-coated silicon nitride windows. Soft X-ray NEXAFS measurements were performed at bending magnet beamline 6.3.2 at the Advanced Light Source under high vacuum (< 1E-6 Torr). X-ray transmission as a function of energy through the adsorbent material was monitored with a photodiode, and a bare polystyrene-coated silicon nitride membrane was used for background correction. For normalization, a line was regressed to the pre-edge region and a polynomial regressed to the post-edge region using the Athena software package.[6]

Near Edge Hard X-ray Absorption (Hard X-ray NEXAFs) and Extended X-ray Absorption (EXAFS)

The Cu K- and Zn K-edge XANES/EXAFS spectra were obtained in transmission mode via a quick scanning EXAFs (QEXAFS) recorder system. By optimizing the oscillating range of the quick-scanning monochromator (quick-mono), the scanning range of photon energy can go up to 2000 eV so that the Cu K- and Zn K-edge spectra can be collected in one scan. All data were carried out with 1 Hz oscillating frequency for 2 min. Two spectra were collected in 1 s, and 240 spectra were averaged to increase the signal-to-noise ratio.

Synchrotron Single Crystal X-ray Diffraction

A single crystal of ZIOS was selected and mounted on Mitegen® loop with Paratone oil. Data were collected on beamline 12.2.1 at the Advanced Light Source using a Bruker D8 diffractometer with a PHOTONII CPAD detector (λ= 0.7288 Å) equipped with an Oxford Cryosystems Cryostream 800 plus, at 100 K. Data reduction and corrections for Lorentz and polarization effects were performed using SAINT[7] v8.38a and were corrected for absorption effects using SADABS v2016/2.[8] Structure solutions were performed by SHELXT[9] using the direct method and were refined by least-square refinement against F^2 by SHELXL.[10] All non-hydrogen atoms were refined anisotropically. For Hydrogen atoms, the –CH– were placed geometrically while the $–CH_3$ were found in the rotation difference map. Both were then constrained and refined with a riding model. The –OH and –NH– hydrogen atoms were found in the difference map and allowed to refine freely.

2. Adsorption tests

Adsorption kinetics for copper capture

~25 mg of ZIF-8 or ZIOS were separately added to conical tubes containing 10 mL of $CuCl_2$ solution (with a copper concentration of 423 ppm, confirmed with ICP-OES) prepared from a 2M $CuCl_2$ stock solution. The samples were then vortexed and sonicated for 1.5 min to obtain well-mixed suspensions. These mixtures were then transferred to a digital tube roller shaker (Cole-Parmer) for a continuous mixing with minimum aeration at a speed of 55 rpm and a 360° degree of rotation, under ambient conditions. At given time points, the mixtures were centrifuged and the supernatants sampled to quantify the remaining copper concentration with ICP-OES. The amount of copper adsorbed by the adsorbents was obtained by subtracting the residual copper concentration from the initial one. Results were then fitted with a pseudo-second-order kinetic model originally proposed by Blanchard[11]:

$$\frac{t}{q_t} = \frac{1}{k_2 q_e^2} + \frac{t}{q_e}$$

where k_2 is the pseudo-second-order rate constant of adsorption (g mg^{-1} min^{-1}) and q_e is the amount of copper ion adsorbed at equilibrium (mg g^{-1}); these values are calculated from the slope and intercept values, respectively, obtained by plotting the linear relationship of (t/q_t) as a function of time, t.[12]

Adsorption isotherms

~2.5 mg of ZIF-8 or ZIOS were separately added to conical tubes containing 6.5 mL of $CuCl_2$ solutions having copper concentrations of 0.45, 2.3, 4.6, 11.5, 23, 46, 103, 207.5, and 415 ppm (confirmed by ICP-OES). The mixtures were then vortexed and sonicated for 1.5 min to obtain well-mixed suspensions. These mixtures were then transferred to a digital tube roller shaker (Cole-Parmer) for continuous mixing

with minimum aeration at a speed of 55 rpm and a 360° degree of rotation, under ambient conditions for 24 h. The mixtures were then centrifuged and the supernatants were analyzed with ICP-OES to quantify the remaining copper content. The amount of copper adsorbed was obtained by subtracting the residual copper concentration from the initial one. The copper uptake data were well-fit with both single-site Langmuir and dual-site Langmuir models (see equations below).[12] Non-linear regressions were used in fitting these models. The fitting parameters and correlation coefficients are summarized in Supplementary Table 1.

Single-site Langmuir model:

$$q_e = \frac{q_{sat} K_L C_e}{1 + K_L C_e}$$

Dual-site Langmuir model:

$$q_e = \frac{q_{sat,1} K_{L,1} C_e}{1 + K_{L,1} C_e} + \frac{q_{sat,2} K_{L,2} C_e}{1 + K_{L,2} C_e}$$

where q_e is the adsorption capacity (mmol g^{-1}), C_e is the equilibrium concentration of the metal ion (mg L^{-1}), q_{sat}, $q_{sat,1}$ and $q_{sat,2}$ are saturation adsorption capacities (mmol g^{-1}) of the adsorption sites, whereas K_L, $K_{L,1}$ and $K_{L,2}$ are Langmuir constants (L mg^{-1}) corresponding to the binding affinities of those adsorption sites.

The Langmuir constant, K_L, obtained from the single-site Langmuir model, was then used to calculate the Gibbs free energy of copper ion adsorption by ZIOS, following the equation below:[13,14]

$$\Delta G^o = -RTlnK_C = -RTln(55.5 * K_L)$$

where ΔG^o is the standard free energy change of the adsorption processes, K_C (dimensionless) is the standard equilibrium constant, 55.5 mol L^{-1} is the calculated value corresponding to the activity of water in solution, which is also equal to its mole concentration. In this equation, K_L is the Langmuir constant (L mol^{-1}), R is gas constant, and T is the temperature at which the adsorption was carried out. The Gibbs free energy of copper adsorption on ZIOS was calculated to be −27.23 kJ.mol^{-1}, which indicates a thermodynamically favorable process.

Furthermore, experimental results from batch adsorption tests were used to calculate the distribution coefficient (K_d) for copper uptake using the equation below:

$$K_d = \frac{C_i - C_f}{C_f} * \frac{V}{m}$$

where C_i is the initial copper ion concentration, C_f is the final equilibrium metal ion concentration, V is the volume of the treated solution (mL) and m is the mass of adsorbent used (g).[5,15] In our experiments, the V/m ratio was set as ~2650 mL g^{-1} at ambient temperature.

Distribution coefficient, K_d. As a measure of the thermodynamic equilibrium of adsorbate / adsorbent binding affinity in aqueous solutions, K_d (mL g^{-1}) is a partition coefficient between the aqueous supernatant phase and the solid adsorbent phase.[15] A high value of K_d indicates the effectiveness of an adsorbent for sequestering the target species from the aqueous solution and the selectivity of the

adsorbent for the target species.[15] Specifically, a K_d > 5000 is considered very good,[15] ≥ 10^4 mL g^{-1} is considered excellent,[16] and > $5*10^4$ is considered outstanding.[12,15] For Cu^{2+}, the high K_d values determined for ZIOS at various Cu^{2+} concentrations (up to 2.3 × 10^5) even when being competed by a wide range of co-existing ions at different pH values (~1.34 × 10^5 at pH 2.45 in an ions mixture), are truly excellent, either matching or outperforming state-of-the-art Cu^{2+} sorbent materials published in literature.[12,15,16,17]

Capability of targeting other ion species and selectivity studies

~2.5 mg of ZIF-8 or ZIOS were separately added to conical tubes containing 10 mL of an aqueous solution having 10 ppm of NaCl, $Ca(NO_3)_2$, $FeCl_2$, $FeCl_3$, $Ni(NO_3)_2$, $MnCl_2$ and $CuCl_2$ co-existing in the system (as determined by ICP-OES). The mixtures were then vortexed and sonicated for 1.5 min to obtain well-mixed suspensions. The pH of the as-prepared ions solution was measured to be 3.94. These mixtures were then transferred to a digital tube roller shaker (Cole-Parmer) for continuous mixing under ambient conditions with minimum aeration at a speed of 55 rpm and 360° degree rotation, for 24 hours. The mixtures were then centrifuged and each supernatant was analyzed with ICP-OES to quantify the remaining metal ion content. The amount of metal ion adsorbed was obtained by subtracting the residual concentration from the initial concentration. The same analysis was performed using similar initial solution having 10 ppm of each co-existing metal ion and an adjusted pH 2.45 (treated with HCl).

3. Water stability test

A few mg of ZIOS were immersed in a centrifuge tube containing 10 mL deionized water and this mixture was sealed and allowed to sit under ambient conditions for 52 d. The water was then removed via centrifuge and decanting, and the solid was rinsed once with deionized water, 3× with methanol, dried at room temperature under vacuum for 20 h, and characterized using powder X-ray diffraction at room temperature (using Bruker AXS D8 Discover GADDS X-Ray Diffractometer equipped with a Vantec-500 area detector and operated at 35kV/40mA with a CoKα wavelength of 1.79 Å).

Supplementary Figures:

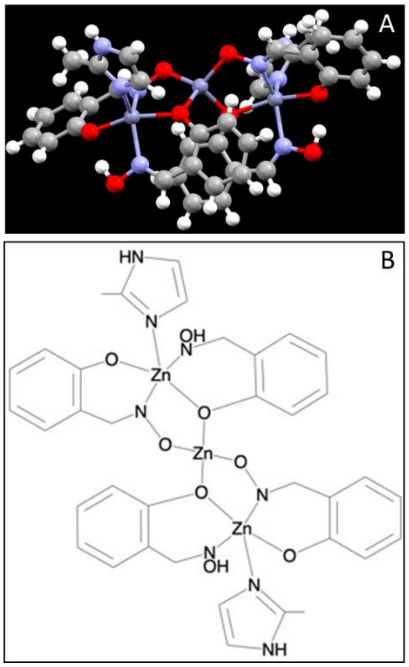

Supplementary Figure 1. Additional view of the structure of a trinuclear unit in ZIOS (A) in ball-and-stick model; and (B) sketched with ChemDraw.

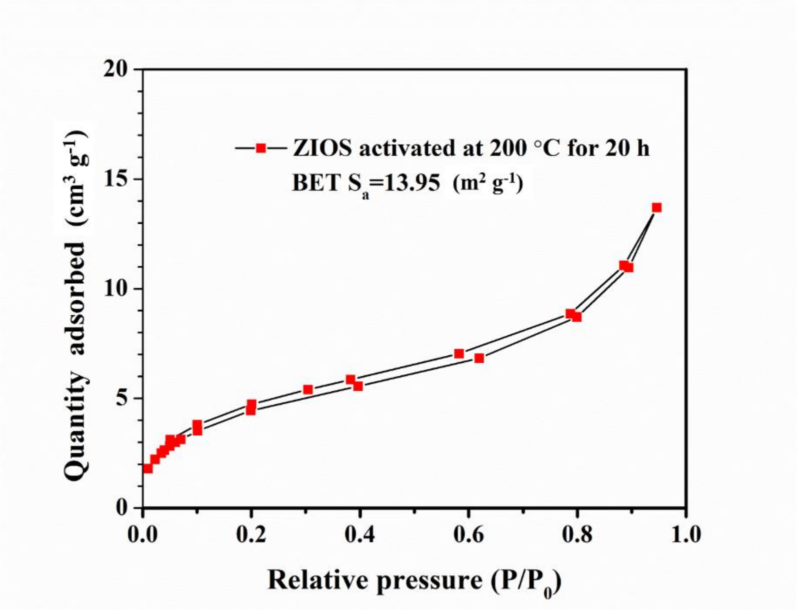

Supplementary Figure 2. N_2 adsorption and desorption isotherms and BET specific surface area of ZIOS after activated at 200 ^oC for 20 hours. The BET surface area of ZIOS measured at this activation condition (200 ^oC) replicates almost exactly that of ZIOS measured after activating the sample at 100 ^oC.

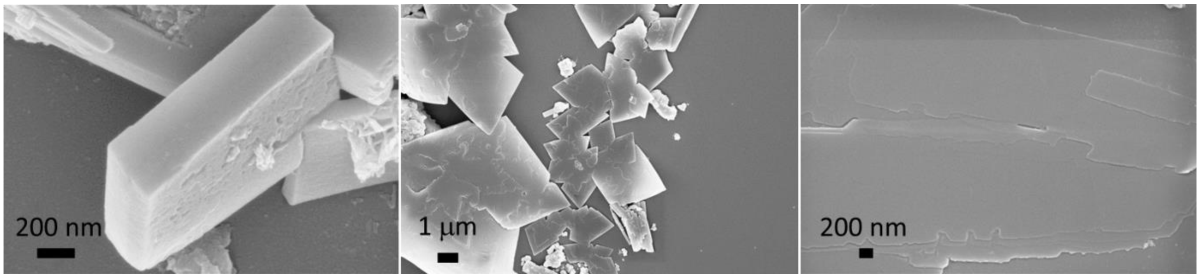

Supplementary Figure 3. Representative scanning electron microscope images of the two-dimensional sheet-like structure of some domains of ZIOS-Cu.

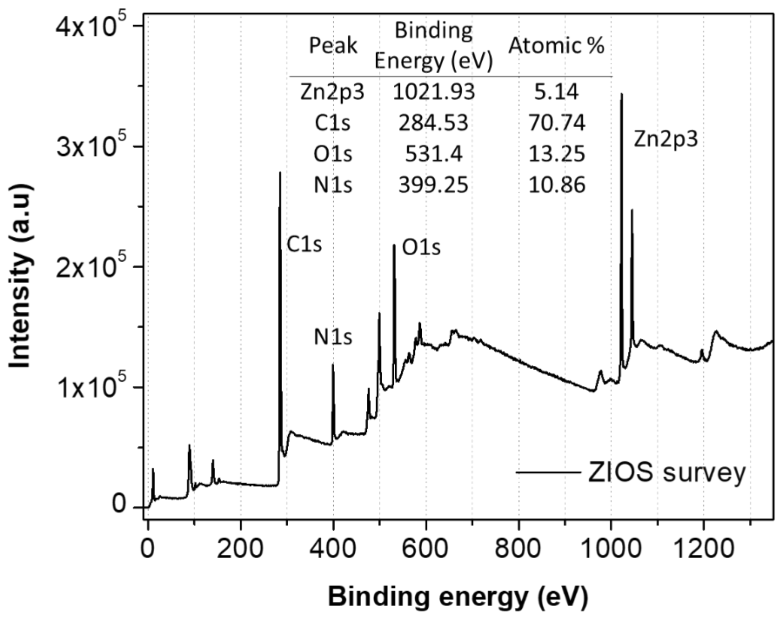

Supplementary Figure 4. XPS survey spectrum of ZIOS.

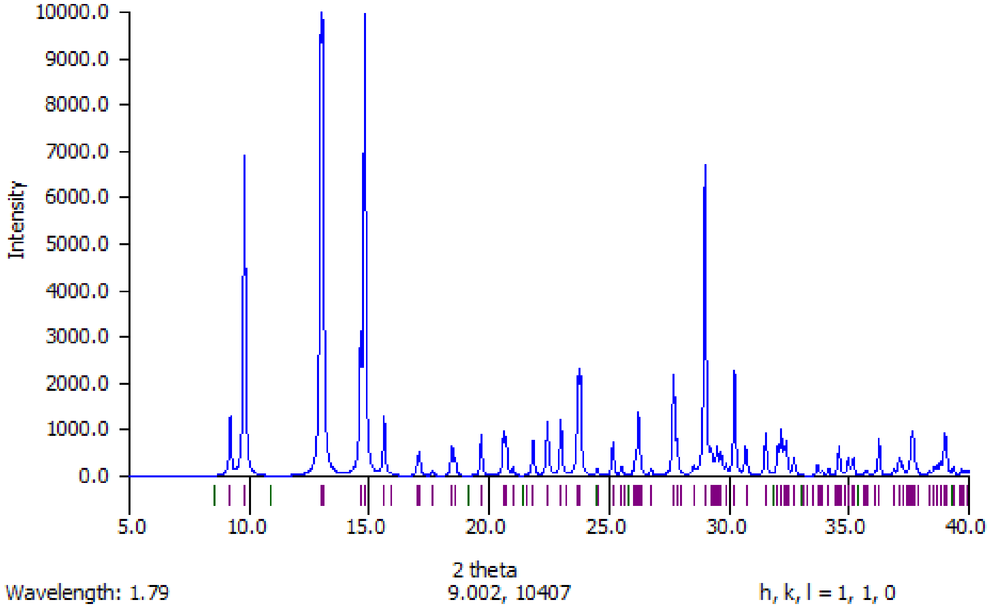

Supplementary Figure 5. Simulated powder X-ray diffraction pattern for ZIOS from single-crystal X-ray diffraction data (Note that the single X-ray diffraction experiment was conducted at 0.7288 Å. However, for the sake of comparison with other spectra collected using powder X-ray diffraction, this pattern was converted to the wavelength of 1.79 Å).

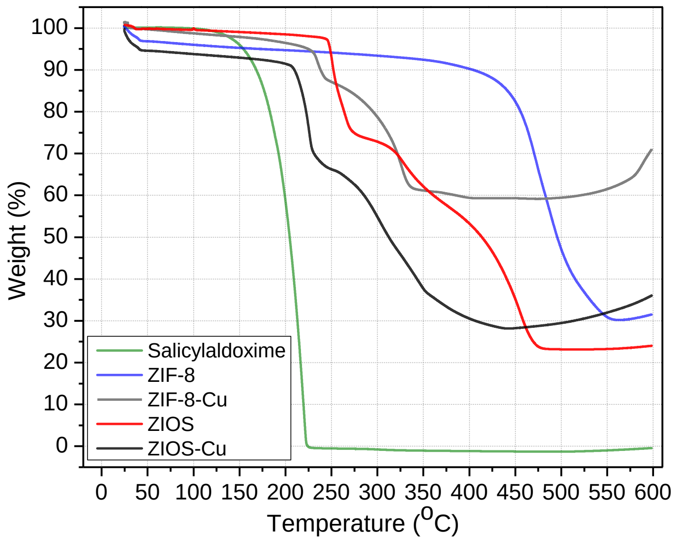

Supplementary Figure 6. TGA profiles of salicylaldoxime (green curve) and ZIF-8 and ZIOS before (blue and red, respectively) and after copper ion adsorption (grey and black, respectively).

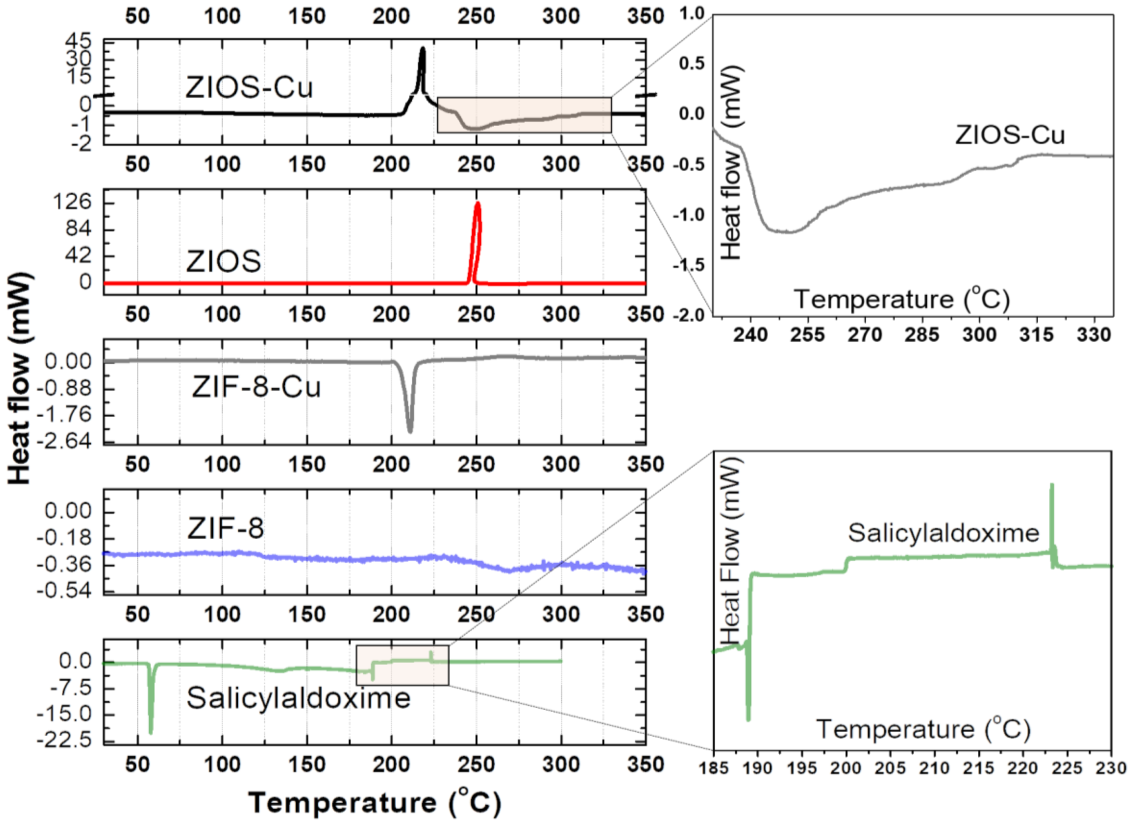

Supplementary Figure 7. DSC profiles of salicylaldoxime (green curve) and ZIF-8 and ZIOS before (blue and red, respectively) and after copper ion adsorption (grey and black, respectively).

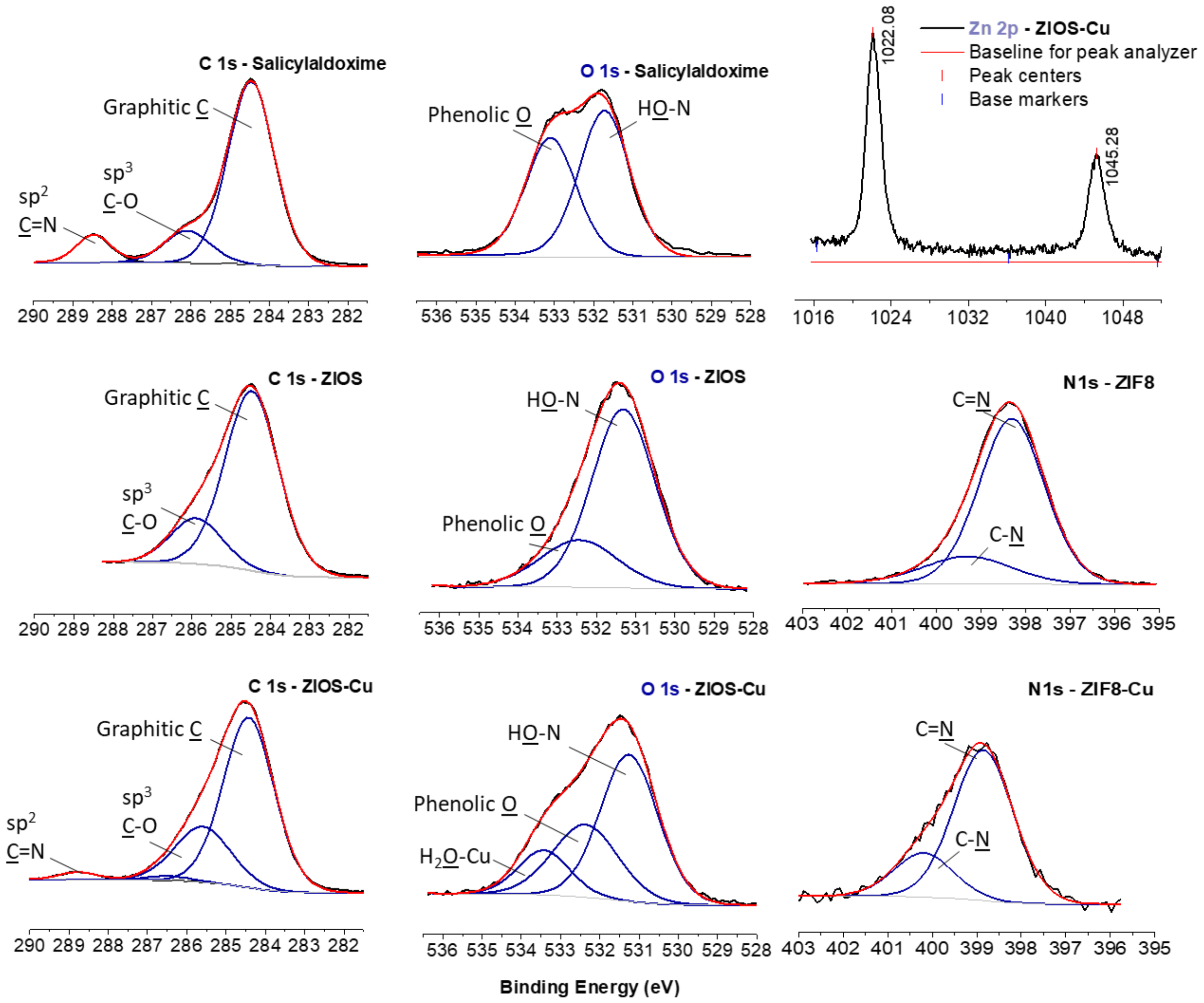

Supplementary Figure 8. X-ray photoelectron binding energy curves showing C 1s (red) and O 1s (blue) energies for ZIOS, ZIOS-Cu, and salicylaldoxime.

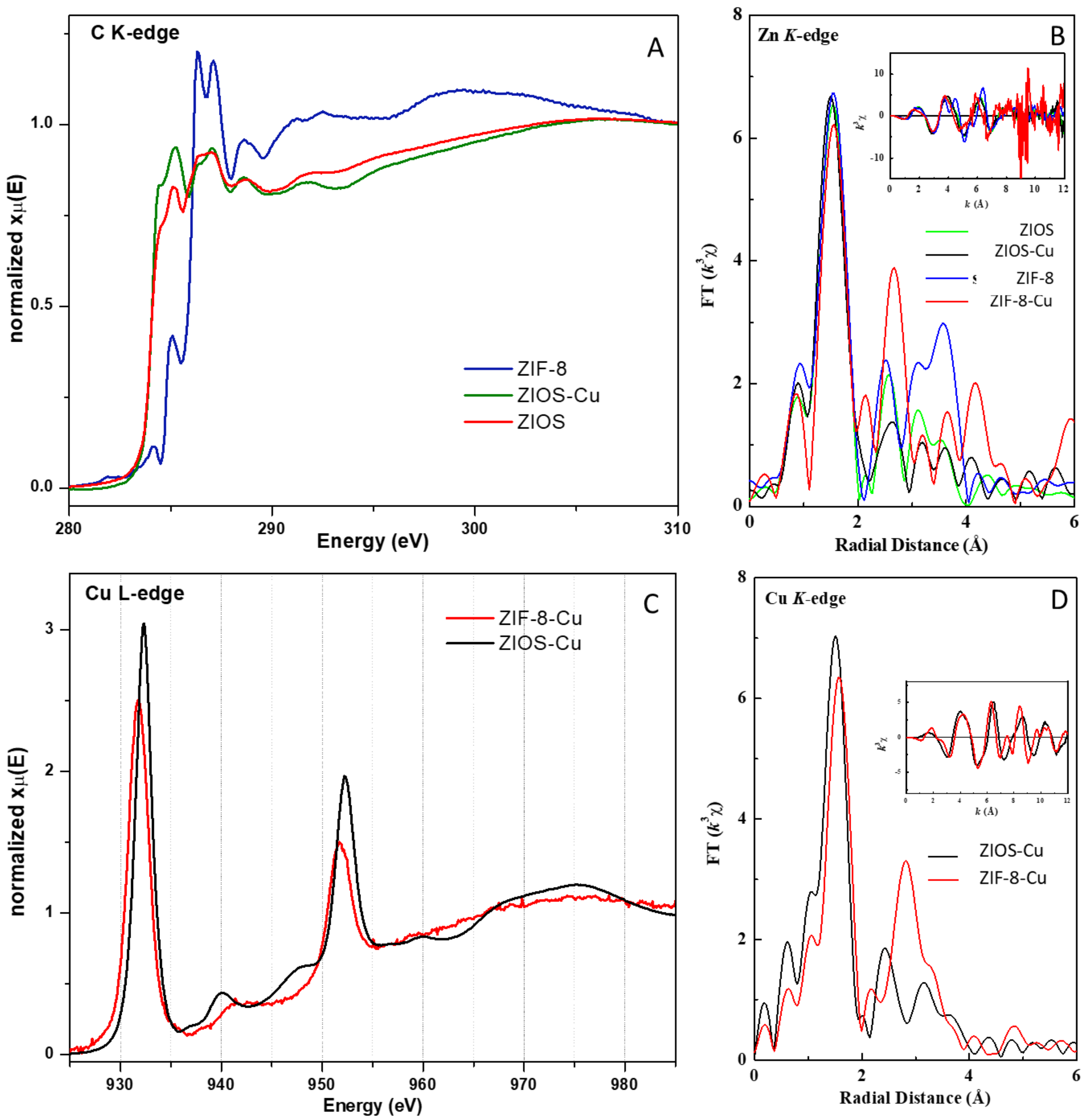

Supplementary Figure 9. NEXAFS profiles near (A) the C K-edge for ZIF-8, ZIOS, and ZIOS-Cu or (C) the Cu L-edge for ZIF-8-Cu and ZIOS-Cu, and experimental Fourier transforms at (B) the Zn K-edge for ZIF-8, ZIOS, and, ZIF-8-Cu, ZIOS-Cu and (D) the Cu K-edge for ZIF-8-Cu and ZIOS-Cu.

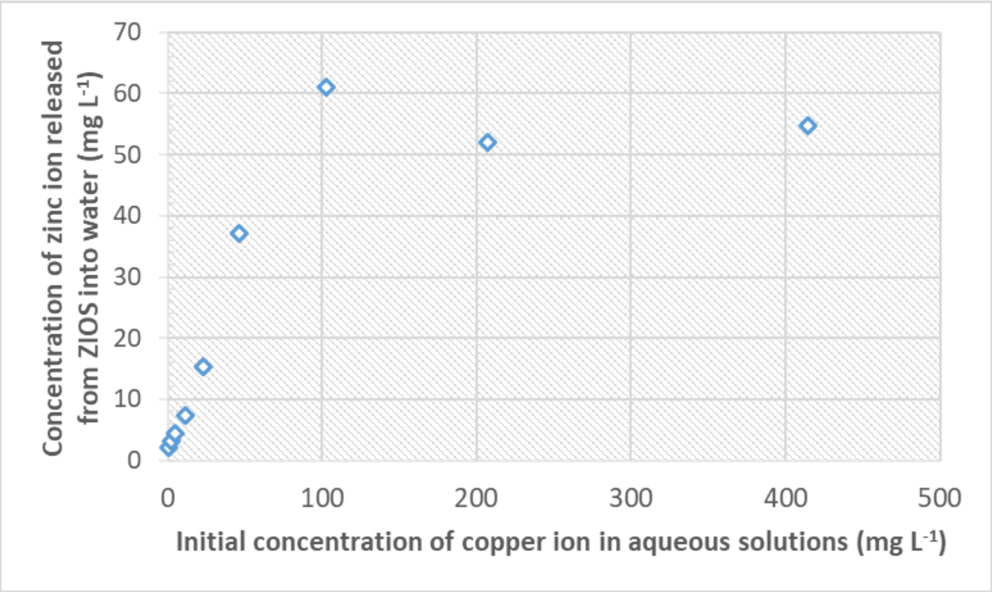

Supplementary Figure 10. The amount of zinc ion released from ZIOS into the water environment measured in the copper adsorption isotherm tests. The initial concentration of copper ion in aqueous solutions ranges from 0.45 to 2.3, 4.6, 11.5, 23, 46, 103, 207.5 and 415 ppm. The ion concentration was measured with ICP-OES.

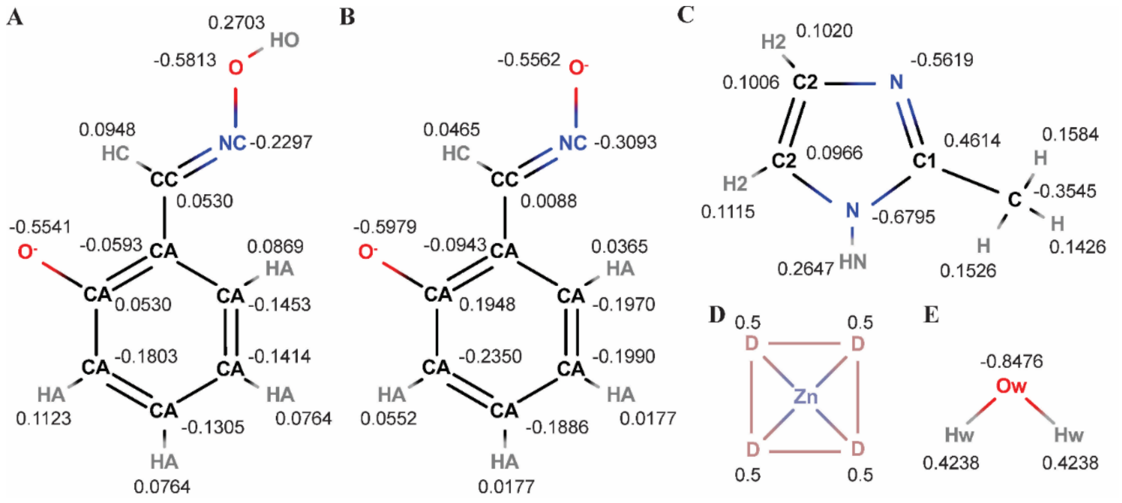

Supplementary Figure 11. Molecular structures and partial atomic charges of (A) oxidized salicylaldoxime (total charge: −1) (B) oxidized salicylaldoxime (total charge: −2) (C) methylimidazole (D) zinc dummy model by cationic dummy atom model and (E) SPC/E water model. The partial atomic charges are used for the *q* parameter in Coulombic force term in Equation 1.

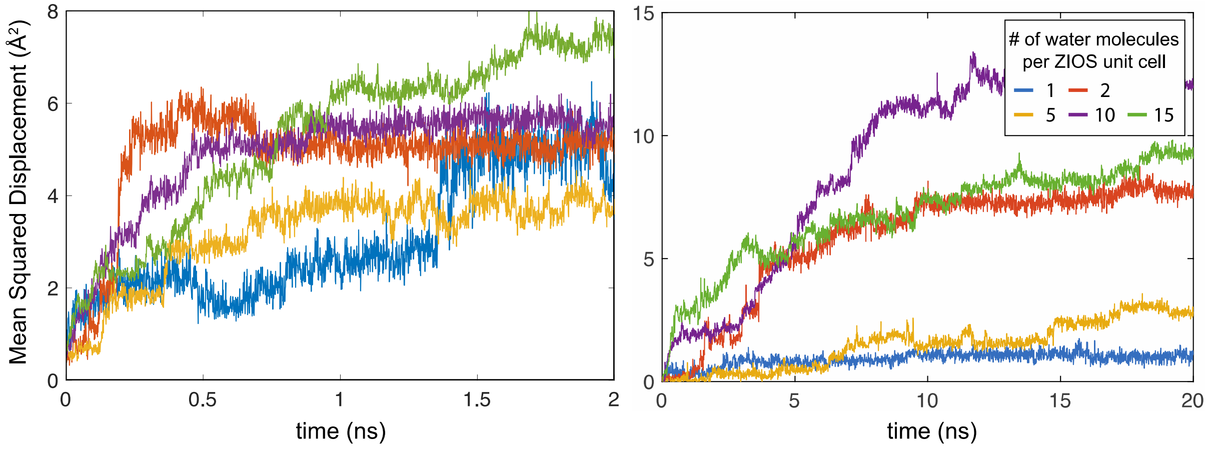

Supplementary Figure 12. Mean squared displacement of water molecules in ZIOS structure before equilibrium (left) and for many ns after equilibrium (right).

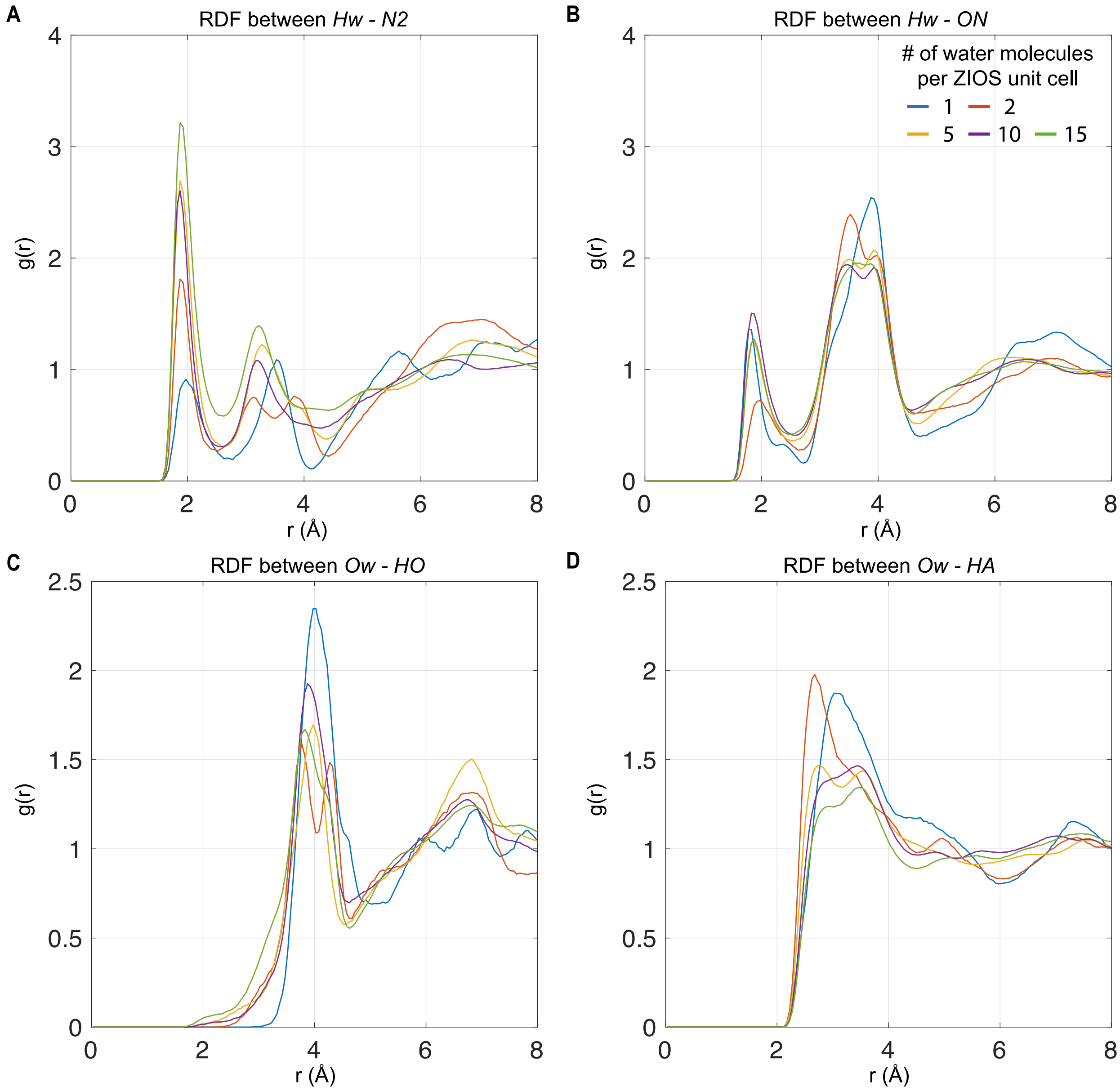

Supplementary Figure 13. Radial Distribution Functions of water molecules from molecular dynamics simulations with different number of water molecules per unit cell. (A) Hydrogen of water molecule hydrogen bonding with N2 atom in ZIOS (B) Hydrogen of water molecule hydrogen bonding with –ON atom in ZIOS (C) Oxygen of water molecule strongly interacting with –HO atom in ZIOS (D) Oxygen of water molecule strongly interacting with –HA atom in ZIOS. (The legend notation is based on the molecular structure given in Supplementary Figure 11).

Supplementary Tables:

Supplementary Table 1. Crystal data and structure refinement for ZIOS.

Identification code	ZIOS	
Empirical formula	C36 H34 N8 O8 Zn3	
Formula weight	902.82	
Temperature	100(2) K	
Wavelength	0.7288 Å	
Crystal system	Monoclinic	
Space group	C2/c	
Unit cell dimensions	a = 19.0510(7) Å	α= 90°.
	b = 15.7429(6) Å	β= 123.7170(10)°.
	c = 14.4822(9) Å	γ = 90°.
Volume	3612.9(3) $Å^3$	
Z	4	
Density (calculated)	1.660 Mg m^{-3}	
Absorption coefficient	2.183 mm^{-1}	
F(000)	1840	
Crystal size	0.020 x 0.010 x 0.010 mm^3	
Theta range for data collection	1.870 to 27.930°.	
Index ranges	-24<=h<=24, -20<=k<=20, -18<=l<=18	
Reflections collected	27325	
Independent reflections	4010 [R(int) = 0.0574]	
Completeness to theta = 25.930°	99.9 %	
Absorption correction	Semi-empirical from equivalents	
Max. and min. transmission	0.979 and 0.834	
Refinement method	Full-matrix least-squares on F^2	
Data / restraints / parameters	4010 / 0 / 258	
Goodness-of-fit on F^2	1.083	
Final R indices [I>2sigma(I)]	R1 = 0.0327, wR2 = 0.0835	
R indices (all data)	R1 = 0.0427, wR2 = 0.0878	
Extinction coefficient	n/a	
Largest diff. peak and hole	0.689 and -0.490 e.$Å^{-3}$	

Supplementary Table 2. Atomic coordinates (x 10^4) and equivalent isotropic displacement parameters (Å^{2}x 10^3)
for ZIOS. U(eq) is defined as one third of the trace of the orthogonalized U^{ij} tensor.

	x	y	z	U(eq)
Zn(1)	5644(1)	7738(1)	5913(1)	28(1)
Zn(2)	5000	8331(1)	7500	28(1)
C(1)	7452(2)	7919(2)	6547(2)	32(1)
O(1)	6689(1)	7596(1)	5889(2)	36(1)
C(2)	8095(2)	7669(2)	6406(2)	39(1)
C(3)	8894(2)	7990(2)	7047(3)	45(1)
C(4)	9105(2)	8562(2)	7885(3)	46(1)
C(5)	8496(2)	8816(2)	8062(2)	38(1)
C(6)	7664(1)	8508(2)	7403(2)	30(1)
C(7)	7081(2)	8808(2)	7679(2)	29(1)
N(1)	6309(1)	8581(1)	7210(2)	28(1)
O(2)	5891(1)	9006(1)	7622(1)	32(1)
C(8)	4248(2)	6879(2)	6124(2)	29(1)
O(3)	4741(1)	7553(1)	6289(1)	29(1)
C(9)	3492(2)	6987(2)	6041(2)	37(1)
C(10)	2995(2)	6297(2)	5926(2)	43(1)
C(11)	3240(2)	5486(2)	5889(2)	45(1)
C(12)	3975(2)	5366(2)	5942(2)	43(1)
C(13)	4485(2)	6050(2)	6044(2)	33(1)
C(14)	5231(2)	5863(2)	6052(2)	41(1)
N(2)	5684(1)	6408(1)	5971(2)	37(1)
O(4)	6349(1)	6053(1)	5956(2)	54(1)
C(15)	5001(2)	9090(2)	4131(2)	29(1)
N(3)	4841(1)	8427(1)	4554(2)	28(1)
C(16)	3972(2)	8373(2)	3987(2)	34(1)
C(17)	3618(2)	8999(2)	3233(2)	37(1)
N(4)	4273(1)	9445(1)	3327(2)	31(1)
C(18)	5845(2)	9412(2)	4477(2)	39(1)

Supplementary Table 3. Bond lengths [Å] and angles [°] for ZIOS.

Zn(1)-N(3)	2.012(2)
Zn(1)-O(1)	2.0225(17)
Zn(1)-N(1)	2.061(2)
Zn(1)-O(3)	2.0938(16)
Zn(1)-N(2)	2.096(2)
Zn(2)-O(2)	1.9251(16)
Zn(2)-O(2)#1	1.9251(16)
Zn(2)-O(3)#1	1.9644(16)
Zn(2)-O(3)	1.9645(16)
C(1)-O(1)	1.320(3)
C(1)-C(2)	1.405(3)
C(1)-C(6)	1.416(4)
C(2)-C(3)	1.365(4)
C(2)-H(2A)	0.9500
C(3)-C(4)	1.379(4)
C(3)-H(3A)	0.9500
C(4)-C(5)	1.379(4)
C(4)-H(4A)	0.9500
C(5)-C(6)	1.407(3)
C(5)-H(5A)	0.9500
C(6)-C(7)	1.451(3)
C(7)-N(1)	1.283(3)
C(7)-H(7A)	0.9500
N(1)-O(2)	1.402(2)
C(8)-O(3)	1.348(3)
C(8)-C(9)	1.389(3)
C(8)-C(13)	1.406(3)
C(9)-C(10)	1.389(4)
C(9)-H(9A)	0.9500
C(10)-C(11)	1.371(4)
C(10)-H(10A)	0.9500
C(11)-C(12)	1.372(4)
C(11)-H(11A)	0.9500
C(12)-C(13)	1.401(4)
C(12)-H(12A)	0.9500
C(13)-C(14)	1.446(4)
C(14)-N(2)	1.268(3)
C(14)-H(14A)	0.9500
N(2)-O(4)	1.395(3)
O(4)-H(4O)	0.95(4)
C(15)-N(3)	1.329(3)
C(15)-N(4)	1.341(3)
C(15)-C(18)	1.483(3)
N(3)-C(16)	1.382(3)

C(16)-C(17)	1.342(4)
C(16)-H(16A)	0.9500
C(17)-N(4)	1.372(3)
C(17)-H(17A)	0.9500
N(4)-H(4N)	0.79(3)
C(18)-H(18A)	0.9800
C(18)-H(18B)	0.9800
C(18)-H(18C)	0.9800

N(3)-Zn(1)-O(1)	105.31(8)
N(3)-Zn(1)-N(1)	107.05(8)
O(1)-Zn(1)-N(1)	88.55(7)
N(3)-Zn(1)-O(3)	92.33(7)
O(1)-Zn(1)-O(3)	161.45(7)
N(1)-Zn(1)-O(3)	91.76(7)
N(3)-Zn(1)-N(2)	124.65(8)
O(1)-Zn(1)-N(2)	82.85(8)
N(1)-Zn(1)-N(2)	128.07(8)
O(3)-Zn(1)-N(2)	82.38(7)
O(2)-Zn(2)-O(2)#1	113.06(10)
O(2)-Zn(2)-O(3)#1	119.33(7)
O(2)#1-Zn(2)-O(3)#1	101.41(7)
O(2)-Zn(2)-O(3)	101.41(7)
O(2)#1-Zn(2)-O(3)	119.33(7)
O(3)#1-Zn(2)-O(3)	102.86(10)
O(1)-C(1)-C(2)	119.1(2)
O(1)-C(1)-C(6)	123.3(2)
C(2)-C(1)-C(6)	117.6(2)
C(1)-O(1)-Zn(1)	130.16(15)
C(3)-C(2)-C(1)	122.0(3)
C(3)-C(2)-H(2A)	119.0
C(1)-C(2)-H(2A)	119.0
C(2)-C(3)-C(4)	120.7(3)
C(2)-C(3)-H(3A)	119.6
C(4)-C(3)-H(3A)	119.6
C(5)-C(4)-C(3)	119.2(3)
C(5)-C(4)-H(4A)	120.4
C(3)-C(4)-H(4A)	120.4
C(4)-C(5)-C(6)	121.5(3)
C(4)-C(5)-H(5A)	119.3
C(6)-C(5)-H(5A)	119.3
C(5)-C(6)-C(1)	119.0(2)
C(5)-C(6)-C(7)	116.6(2)
C(1)-C(6)-C(7)	124.4(2)
N(1)-C(7)-C(6)	126.3(2)
N(1)-C(7)-H(7A)	116.9

C(6)-C(7)-H(7A)	116.9
C(7)-N(1)-O(2)	113.44(19)
C(7)-N(1)-Zn(1)	127.24(16)
O(2)-N(1)-Zn(1)	119.24(13)
N(1)-O(2)-Zn(2)	112.22(13)
O(3)-C(8)-C(9)	120.4(2)
O(3)-C(8)-C(13)	121.5(2)
C(9)-C(8)-C(13)	118.1(2)
C(8)-O(3)-Zn(2)	117.07(14)
C(8)-O(3)-Zn(1)	130.95(15)
Zn(2)-O(3)-Zn(1)	108.03(7)
C(10)-C(9)-C(8)	121.3(3)
C(10)-C(9)-H(9A)	119.3
C(8)-C(9)-H(9A)	119.3
C(11)-C(10)-C(9)	120.6(3)
C(11)-C(10)-H(10A)	119.7
C(9)-C(10)-H(10A)	119.7
C(10)-C(11)-C(12)	119.1(3)
C(10)-C(11)-H(11A)	120.5
C(12)-C(11)-H(11A)	120.5
C(11)-C(12)-C(13)	121.7(3)
C(11)-C(12)-H(12A)	119.1
C(13)-C(12)-H(12A)	119.1
C(12)-C(13)-C(8)	119.1(2)
C(12)-C(13)-C(14)	117.7(2)
C(8)-C(13)-C(14)	123.2(2)
N(2)-C(14)-C(13)	125.3(2)
N(2)-C(14)-H(14A)	117.3
C(13)-C(14)-H(14A)	117.3
C(14)-N(2)-O(4)	113.6(2)
C(14)-N(2)-Zn(1)	131.97(18)
O(4)-N(2)-Zn(1)	114.38(16)
N(2)-O(4)-H(4O)	94(2)
N(3)-C(15)-N(4)	109.6(2)
N(3)-C(15)-C(18)	126.6(2)
N(4)-C(15)-C(18)	123.8(2)
C(15)-N(3)-C(16)	106.26(19)
C(15)-N(3)-Zn(1)	129.03(16)
C(16)-N(3)-Zn(1)	123.87(16)
C(17)-C(16)-N(3)	109.5(2)
C(17)-C(16)-H(16A)	125.2
N(3)-C(16)-H(16A)	125.2
C(16)-C(17)-N(4)	106.0(2)
C(16)-C(17)-H(17A)	127.0
N(4)-C(17)-H(17A)	127.0
C(15)-N(4)-C(17)	108.6(2)

C(15)-N(4)-H(4N)	123.5(19)
C(17)-N(4)-H(4N)	127.7(19)
C(15)-C(18)-H(18A)	109.5
C(15)-C(18)-H(18B)	109.5
H(18A)-C(18)-H(18B)	109.5
C(15)-C(18)-H(18C)	109.5
H(18A)-C(18)-H(18C)	109.5
H(18B)-C(18)-H(18C)	109.5

Symmetry transformations used to generate equivalent atoms:
#1 -x+1,y,-z+3/2

Supplementary Table 4. Pseudo-second-order rate constant of adsorption, k_2 (g mg^{-1}min^{-1}), the amount of copper ion adsorbed at equilibrium, q_e (mg g^{-1}), and the saturation capacity, q_{sat} (mg g^{-1}), obtained from Langmuir fits to adsorption isotherms.

**Materials**	k_2 (mg mg^{-1} min^{-1})		q_e (mg g^{-1})	q_{sat} (mg g^{-1})
ZIF-8 *(this study)	3.4 ± 1.6	(x)	116.4 ± 5.7	-
ZIOS (this study)	**155.3 ± 26.5**	**(46x)**	162.9 ± 9.4	208.5
PAF-1-SMe* [1]	5.2	(~ x)	-	662
Ca-MOF [2]	32 ± 5	(9x)	-	68
Metal sulfide ion exchangers [3]	5.8 – 23.9	(x – 7x)	-	155
Commercially available thiol-functionalized resin (Duolite GT73) [1,4]	-		25 at 160 ppm Cu^{2+}	62.1
Amberlite IRC-718 (active group: iminodiacetic acid)** [4]	-		-	128
Zeolites, biomass-derived adsorbents [1]		≤ x	-	5.1 – 133.4
Silica-based polymers [1]		≤ x	-	0.5 – 147

* ZIF-8 and PAF-1-Sme are current state-of-the-art Cu^{2+} adsorbents
** one of the commercial resins that has the highest adsorption for Cu, Cd, Ni and Zn

Supplementary Table 5. Parameters from fitting the empirical adsorption isotherm of ZIOS with different adsorption models.

Model	Freundlich	Single-site Langmuir	Dual-site Langmuir
Parameters	K_F = 12.32 $mg^{1-(1/n)}$ $L^{1/n}$ g^{-1}<br>n = 2.20	q_{sat} = 208.44 mg g^{-1}<br>K_L = 0.0167 L mg^{-1}	$q_{sat,1}$ = 1.62 mg g^{-1}<br>$K_{L,1}$ = 0.0167 L mg^{-1}<br>$q_{sat,2}$ = 206.83 mg g^{-1}<br>$K_{L,2}$ = 0.0167 L mg^{-1}
R^2	0.9473	0.9987	0.9978

Supplementary Table 6. Copper selectivity of ZIOS when exposed to aqueous solutions simultaneously containing equimolar concentration (~ 10 ppm) of Na^+, Ca^{2+}, Fe^{3+}, Ni^{2+}, Cu^{2+}, and ~30 ppm of Zn^{2+}.

An aqueous solution having 10 ppm of Na+, Ca^{2+}, Fe^{3+}, Ni^{2+}, and Cu^{2+}, co-existing with 30 ppm Zn^{2+}				
		Cu^{2+}	Ni^{2+}	Zn^{2+}
Before adsorption		9.5 ± 1.2	12.4 ± 0.7	32.1 ± 1.9
After 24-hr adsorption	as-prepared	0*	0*	21.8 ± 0.3
	pH was adjusted to 2.48	1.2 ± 0.2	13.5 ± 0.02	48.9 ± 2.3
		*undetectable		

The initial concentration ratio of Cu^{2+}: Zn^{2+} is 1:3. As Ni^{2+}, Cu^{2+}, and Zn^{2+} are the three ions of interest (based on the previous selectivity test without the presence of Zn^{2+}), we tracked the concentration of these three cations before and after adsorption with ICP-OES. Below is the table showing the concentration of these ions before and after adsorption. Results show that the copper selectivity of ZIOS in an aqueous environment having Zn^{2+} at three times more concentrated (e.g. Cu^{2+}: Zn^{2+} = 1:3) remained unchanged. Interestingly, at pH 2.48, the Cu/Ni selectivity behavior of ZIOS is again observed. ZIOS appears to not uptake Ni^{2+} at this pH value. Meanwhile, at as-prepared pH, ZIOS appears to have some capacity to further uptake Zn^{2+} presenting in the solution on top of Cu^{2+} and Ni^{2+}.

Supplementary Table 7. Lennard-Jones 12-6 potential parameters used in molecular dynamics simulations (refer to Supplementary Fig. 11 for atom labels).

molecule	atoms	σ (Å)	ε(kcal mol^{-1})	source
methylimidazole	CA	3.5465	0.0700	[18]
	CC	3.5600	0.1100	[18]
	NC	3.2930	0.2000	[18]
	HA	2.3496	0.0220	[18]
	HC	2.3496	0.0220	[18]
	HO	0.3996	0.0460	[18]
	O	3.1506	0.1521	[18]
salicylaldoxime	C1	3.4000	0.0860	[19]
	C2	3.4000	0.0860	[19]
	C	3.4000	0.1094	[19]
	N	3.2500	0.17	[19]
	H	2.4710	0.0157	[19]
	H2	2.5110	0.0150	[19]
	HN	1.2000	0.0157	AMBER
Zinc dummy model	Zn	1.9500	0.2500	[20]
	D	0	0	[20]
water	OW	3.1660	0.1553	[21]
	HW	0	0	[21]

Supplementary Table 8. Bond parameters for the harmonic potential force field used in molecular dynamics simulations and average bond values from molecular dynamics simulation runs.

molecule	bonds	r_0 (Å)	K_r (kcal mol^{-1} $Å^{-2}$)	source	r_{Result} (Å)
methylimidazole	CA-CA	1.374	401.068	[18]	1.387
	CA-CC	1.457	322.985	[18]	1.473
	CA-HA	1.084	381.853	[18]	1.084
	CA-O	1.376	404.019	[18]	1.386
	CC-HC	1.101	334.643	[18]	1.103
	CC-NC	1.290	725.204	[18]	1.294
	O-NC	1.395	323.200	[18]	1.375
	O-HO	0.972	560.905	[18]	0.988
salicylaldoxime	C1-C	1.490	346.543	[19]	1.471
	C2-C2	1.350	540.249	[19]	1.348
	C2-H2	1.080	367.000	[19]	1.082
	C-H	1.090	340.000	[19]	1.088
	N-C1	1.335	488.000	[19]	1.328
	N-C2	1.370	440.210	[19]	1.375
	N-HN	1.010	434.000	AMBER	1.012
Zinc dummy model	Zn-D	0.900	640.000	[20]	-
	D-D	1.470	640.000	[20]	-

Supplementary Table 9. Angle parameters for the harmonic potential force field used in molecular dynamics simulations and average angle values from molecular dynamics simulation run

molecule	angles	θ_0 (deg)	K_θ (kcal mol^{-1} rad^{-2})	source	θ_{Result} (deg)
methylimidazole	CA-CA-CA	120.00	48.150	[18]	119.94
	CA-CA-CC	114.50	57.430	[18]	120.06
	CA-CA-O	116.50	69.660	[18]	119.57
	CA-CA-HA	120.60	40.520	[18]	119.97
	CA-CC-HC	116.40	40.590	[18]	115.51
	CA-CC-NC	119.60	71.750	[18]	121.97
	CC-NC-O	106.90	113.630	[18]	108.66
	NC-CC-HC	119.50	44.840	[18]	120.87
	NC-O-HO	101.60	80.240	[18]	102.53
salicylaldoxime	C1-C-H	109.32	48.088	[19]	109.44
	C1-N-C2	105.27	71.254	[19]	106.48
	C1-N-HN	120.00	50.000	AMBER	125.46
	C2-C2-H2	125.67	49.451	[19]	125.29
	C2-C2-N	108.65	73.750	[19]	107.80
	C2-N-HN	120.00	50.000	AMBER	126.28
	C-C1-N	123.92	66.015	[19]	124.37
	N-C1-N	112.16	75.484	[19]	111.05
	N-C2-H2	125.68	49.954	[19]	126.82
	H-C-H	109.50	35.000	[19]	109.43
Zinc dummy model	Zn-D-D	35.250	55.000	[21]	-
	D-Zn-D	109.50	55.000	[21]	-
	D-D-D	60.00	55.000	[21]	-

Supplementary Table 10. Proper torsion parameters for the force field used in molecular dynamics simulations.

molecule	torsions	ɣ (deg)	K_{ϕ} (kcal mol^{-1})	n	source
methylimidazole	X-CA-CA-X	180	3.50	2	[18]
	X-CA-CC-X	180	1.30	2	[18]
	O-NC-CC-HC	180	8.00	2	[18]
	O-NC-CC-CA	180	0.90	2	[18]
	HO-O-NC-CC	180	1.80	2	[18]
salicylaldoxime	X-C1-N-X	180	2.33	2	[19]
	X-C2-N-X	180	5.00	2	[19]
	X-C2-C2-X	180	5.15	2	[19]
	X-X-C1-C	0	0	2	[19]
	Zn-D-D-D	35	0	2	[21]
	D-Zn-D-D	120	0	2	[21]
	D-D-D-D	71	0	2	[21]

Supplementary Table 11. Improper torsion parameters for the harmonic potential force field used in molecular dynamics simulations.

molecule	improper	χ_0 (deg)	K_{χ} (kcal mol^{-1} rad^{-2})	source
methylimidazole	CA-CA-CA-HA	180	1.079	[18]
	CA-CA-CA-O	180	3.454	[18]
	CA-CA-CA-CC	180	1.943	[18]
	CA-NC-CC-HC	180	9.356	[18]
salicylaldoxime	N-C-C1-N	180	2	[19]
	C2-H2-C2-N	180	2	[19]
	C2-H2-C-N	180	2	[19]
	C1-HN-N-C2	180	2	[19]

Supplementary References

1. Gagné, R. R. *et al.* X-Ray photoelectron spectra of copper(I) and copper(II) complexes derived from macrocyclic ligands. *J. Am. Chem. Soc.* **102**, 1905–1909 (1980).

2. Fadley, C. S. & Shirley, D. A. Multiplet splitting of metal-atom electron binding energies. *Phys. Rev. A - Gen. Phys.* **2**, 1109–1120 (1970).

3. Ávila-Torres, Y., Huerta, L. & Barba-Behrens, N. XPS-characterization of heterometallic coordination compounds with optically active ligands. *J. Chem.* **2013**, (2013).

4. Akshaya, K. B. *et al.* Trace level determination of Hg^{2+} ions in environmental samples with a mercaptotriazole-functionalized TiO_2 nanostructure-based fluorescent probe. *Anal. Methods* **11**, 537–547 (2019).

5. Walton, K. S. & Snurr, R. Q. Applicability of the BET method for determining surface areas of microporous metal-organic frameworks. *J. Am. Chem. Soc.* **129**, 8552–8556 (2007).

6. Ravel, B. & Newville, M. ATHENA, ARTEMIS, HEPHAESTUS: Data analysis for X-ray absorption spectroscopy using IFEFFIT. *J. Synchrotron Radiat.* **12**, 537–541 (2005).

7. Bruker AXS Inc. SAINT Software for CCD Diffractometers, Madison, WI, 2014.

8. Sheldrick, G.M., S. Bruker Analytical X-ray Systems, Inc., Madison, WI, 2000.

9. Sheldrick, G. M. Crystal structure refinement with SHELXL. *Acta Crystallogr. Sect. C, Struct. Chem.* **71**, 3–8 (2015).

10. Sheldrick, G. M. A short history of SHELX. *Acta Crystallogr. A.* **64**, 112–22 (2008).

11. Blanchard, G., Maunaye, M. & Martin, G. Removal of heavy metals from waters by means of natural zeolites. *Water Res.* **18**, 1501–1507 (1984).

12. Lee, S. *et al.* Copper capture in a thioether-functionalized porous polymer applied to the detection of Wilson's disease. *J. Am. Chem. Soc.* **138**, 7603–7609 (2016).

13. Zhou, X. & Zhou, X. The unit problem in the thermodynamic calculation of adsorption using the Langmuir equation. *Chem. Eng. Commun.* **201**, 1459–1467 (2014).

14. Nguyen, H., You, S. & Hosseini-bandegharaei, A. Mistakes and inconsistencies regarding adsorption of contaminants from aqueous solutions : A critical review. *Water Res.* **120**, 88–116 (2017).

15. Fryxell, G. E., Lin, Y., Fiskum, S., Birnbaum, J. C. & Wu, H. Actinide sequestration using self-assembled monolayers on mesoporous supports. **39**, 1324–1331 (2005).

16. Manos, M. J. & Kanatzidis, M. G. Metal sulfide ion exchangers: superior sorbents for the capture of toxic and nuclear waste-related metal ions. *Chem. Sci.* **7**, 4804–4824 (2016).

17. Ma, S. *et al.* Highly selective and efficient heavy metal capture with polysulfide intercalated layered double hydroxides. *J. Mater. Chem. A* **2**, 10280–10289 (2014).

18. Fatehi, M., Mohebbi, A. & Moradi, A. Understanding the structural, dynamic and thermodynamic properties of 5-Nonylsalicylaldoxime: Molecular dynamics and experimental studies. *J. Mol. Liq.* **271**, 290–300 (2018).

19. Zheng, B., Sant, M., Demontis, P. & Suffritti, G. B. Force field for molecular dynamics computations in flexible ZIF-8 framework. *J. Phys. Chem. C* **116**, 933–938 (2012).

20. Biswal, D. & Kusalik, P. G. Molecular simulations of self-assembly processes in metal-organic frameworks: Model dependence. *J. Chem. Phys.* **147**, (2017).

21. Pang, Y.-P. Novel Zinc Protein Molecular Dynamics Simulations: Steps toward antiangiogenesis for cancer treatment. *J. Mol. Model.* **5**, 196–202 (2002).
