# Supplementary material for: A nature-inspired hydrogen-bonded supramolecular complex for selective copper ion removal from water
Source: Nat Commun. 2020 Aug 7;11:3947. doi: 10.1038/s41467-020-17757-6 (PMC7415137; doi:10.1038/s41467-020-17757-6)
Supplement: Supplementary file 2 — Peer Review File [file 41467_2020_17757_MOESM2_ESM.docx]

Peer Review Information

**Journal:** Nature Communications

**Manuscript title:** A nature-inspired hydrogen-bonded supramolecular complex for selective copper ion removal from water

**Corresponding author name(s):** Dr Jeffery Urban

**Editorial notes:**

**Transferred manuscripts**

This manuscript has been previously reviewed at another journal that is not operating a transparent peer review scheme. This document only contains reviewer comments and rebuttal letters for versions considered at *Nature Communications*.

**Reviewer comments & decisions:**

**Reviewer comments, first version:**

Reviewer #1 (Remarks to the Author):

The manuscript by Bui, Kang and coworkers describes the synthesis of a new, water stable hydrogen-bonded inorganic-organic material (ZIOS) and the application of this material in the removal of Cu2+ from water. The maximum capacity for Cu2+ in ZIOS was found to be higher than that of benchmark materials ZIF-8, and Amberlite. The manuscript is well-written and contains all details required to reproduce the study. The characterization of ZIOS is thorough, and the state-of-the-art synchrotron techniques are used to understand the mechanism of Cu2+ adsorption in ZIOS (as well as in the ZIF-8 control sample) which unambiguously confirms how Cu2+ interacts with the materials. Interestingly, some of the results contradict earlier work by Zhang and coworkers (Dalton Trans) regarding Cu2+ uptake in ZIF-8, meaning that this work also represents an important correction to the literature in the field of MOFs.
Some minor revisions listed below would help to clarify a few points:
1. In lines 66-72, the authors should expand the discussion about why removing copper from water is important. They say it’s toxic to humans in “large” quantities – but some values should be given here. When they say it impacts heart development, do they mean in unborn babies? Some examples of the types of industries where copper removal is important should also be included. Later the authors discuss acid main drainage, and also Ni/Cu separation, but these potential applications appear out of nowhere and should be discussed in the intro section as well.
2. On line 84, the authors mention “pH ranges of interest” for copper removal applications. There should be more discussion about these pH ranges of interest and the specific copper removal applications that correspond to specific pH.
3. In Figure 1, the authors should also include ChemDraw images of the linkers since it’s hard to view them in the crystallographic pictures
4. The authors suggest that ZIOS is not porous, but from the experimental section, it seems only one activation condition was attempted (100C for 20 hours). Did the authors try any other conditions to activate ZIOS? For a newly reported material, more than one attempt at activation should be performed before suggesting the material is not porous. Alternatively, the theoretical surface area should be calculated using GCMC simulations and compared to the experimental value obtained.
5. Is there a reason for choosing 895ppm (or 425ppm Cu2+) concentration as a starting point? If so, that should be mentioned.
6. On line 249 (and later), the authors suggest the possibility of transmetalation. ICP-OES should be performed to determine if Zn is present (and at what concentrations) in the water samples being treated. The later ICP data (line 334-335) doesn’t really give this information since the theoretical wt% of Zn in the samples before and after adsorption of Cu is not given.
7. On lines 296-299, the authors discuss how their results contradict previous reports on Cu adsorption and metal-exchange. Are the studies performed in this manuscript done at the same concentrations as those performed previously? The concentrations (Cu:MOF ratio) in this study vs. representative previous studies should be included.


Reviewer #2 (Remarks to the Author):

The manuscript from Bui et al. describes their investigation of a novel material for the selective capture of copper ions from water. This material is a framework structure comprised of hydrogen-bonded supramolecular complexes of zinc, 2-methylimidazole and salicylaldoxime (ZIOS). In this submission, the authors study the copper uptake of ZIOS and compare it the metal-organic framework ZIF-8, which has a similar framework composition but lacks the salicylaldoxime functionality. ZIOS, unlike ZIF-8, displayed excellent copper uptake, kinetics and meaningful selectivity thus highlighting this material as a state-of-the-art adsorbent for copper in water. Subsequently, to provide insight to the mechanism responsible for this impressive uptake, adsorption X-ray photoelectron spectroscopy (XPS), synchrotron radiation near-edge X-ray absorption near-edge structure (NEXAFS) spectroscopy and extended X-ray absorption fine structure (EXAFS) spectroscopy were employed to characterise the materials before and after copper adsorption.
This comprehensive experimental analysis identified the salicylaldoxime and imidazole ligands, in ZIOS, are involved in copper coordination, and there is some degree or chemisorption, while ZIF-8 shows the complete transformation of environments, in agreement with the powder X-ray diffraction pattern. Finally, classical molecular simulations were performed to understand the impressive uptake kinetics. The simulations demonstrate a reversible increase in the lattice constant and step-wise diffusion of water, which is considered responsible for the fast adsorption of zinc in this formally non-porous structure.

I believe the quality and potential influence of this work is commensurate with the quality expected by readers of Nature Communications and is publishable subject to revision, following the comments raised below.

1. This article is mostly well written and very enjoyable read, however, the description of X-ray spectroscopy characterisation was overly complex and ultimately I am unsure what of value can be inferred from this. I would suggest significantly shortening this section and highlighting concisely what insight this analysis provides.

2. The transformations of ZIF-8 in an aqueous copper environment are not overly surprising given the propensity for biomolecule@ZIF-8 phase changes in response to post-synthesis treatments (e.g. washing ) [10.1021/acs.chemmater.7b04977, 10.1039/c8mh01611a]. The mechanism present in these studies is also likely to be present here, and responsible for the crystal-to-crystal transformation observed.

3. As the crystal morphology is not overly changed by the process of Cu adsorption, and good crystallinity is present, were single-crystal X-ray diffraction experiments performed? As this would unequivocally show the location of Cu.

4. It is stated that transmetalation in ZIOS does occur, evidenced by the leaching of Zn after adsorption. How problematic is this for the application of the material? Moreover, was the selectivity of Cu in a mixture containing Zn investigated?

5. The breathing effect demonstrated by molecular simulation, while interesting, is hardly comparable to the experimental system. Firstly, the molecular simulations show behaviour reminiscent of the swelling of clay structures [10.1063/1.1493186]. Although it is stated that the hopping of water molecules goes from hydrogen bonding sites there is no evidence of this reported. It could just as likely be sequential hydration of interlayer sites, as the material is indeed a two-dimensional framework. The radial distribution functions (RDF) display does not provide sufficient evidence for H-bonding sites. For example, the hydrogen atoms of the benzene group are unlikely to be involved in hydrogen bonding. The methodology behind the calculation of the hopping rate is not presented and I would be hesitant to compare this directly to Aquaporin-1. Furthermore, this swelling mechanism (I would refrain from using the term breathing as this is often associated with a non-monotonic volume change upon adsorption) is not observed experimentally, despite the comprehensive experimental characterisation, why?

**Author rebuttal, first version:**

**Reviewer #1 (Remarks to the Author):**

The manuscript by Bui, Kang and coworkers describes the synthesis of a new, water stable hydrogen-bonded inorganic-organic material (ZIOS) and the application of this material in the removal of Cu2+ from water. The maximum capacity for Cu2+ in ZIOS was found to be higher than that of benchmark materials ZIF-8, and Amberlite. The manuscript is well-written and contains all details required to reproduce the study. The characterization of ZIOS is thorough, and the state-of-the-art synchrotron techniques are used to understand the mechanism of Cu2+ adsorption in ZIOS (as well as in the ZIF-8 control sample) which unambiguously confirms how Cu2+ interacts with the materials. Interestingly, some of the results contradict earlier work by Zhang and coworkers (Dalton Trans) regarding Cu2+ uptake in ZIF-8, meaning that this work also represents an important correction to the literature in the field of MOFs. Some minor revisions listed below would help to clarify a few points:

We thank the Reviewer for the thorough review and positive comments on the merit and impacts of the manuscript. Thanks also for the valuable suggestions as minor revisions which help clarify our work. Please see below for the details of our revision.

1. In lines 66-72, the authors should expand the discussion about why removing copper from water is important. They say it’s toxic to humans in “large” quantities – but some values should be given here. When they say it impacts heart development, do they mean in unborn babies? Some examples of the types of industries where copper removal is important should also be included. Later the authors discuss acid main drainage, and also Ni/Cu separation, but these potential applications appear out of nowhere and should be discussed in the intro section as well.

We thank the Reviewer for this good point. We have updated the manuscript to include more specific values regarding copper toxicity. We also changed the language from “heart development” to “heart function” to keep our discussion on a more general level. Furthermore, examples of the types of industries where copper removal is important were included as suggested. Copper removal from acid mine drainage and Ni/Cu separation was briefly included in the introduction. Please see below for the details.

The revised text is below and can be found in the main text (annotated version): pages 2-3, lines 57-97:

“**Copper, one of the most widely used heavy metals, is toxic to humans in large quantities, despite its essential role in our physiology.^4^ A copper imbalance can impact heart function and lipid metabolism as well as causing inflammation and resistance to chemotherapeutic drugs.^5^ The U.S. Environmental Protection Agency has established a maximum level of copper in drinking water at approximately 1.3 mg/L.^7^ However, copper levels reported in drinking water in Europe, Canada and the USA can range from ≤ 0.005 to > 30 mg/L, originating primarily from the corrosion of interior copper plumping.^7^Historically, there have been several outbreaks of copper poisoning from ingestion of beverages that were contaminated with copper salts deposited in the original water sources.^6^ Given that millions of tons of copper are produced annually worldwide and it is ubiquitous across industries,^9^ maintaining low levels of copper contaminants in water streams is a present concern. Therefore, seeking highly-efficient approaches for copper removal from water is important. Beyond human health, selective separations involving copper is a challenging and relevant area of research for which traditional metallurgical solutions are limited. In mineral processing activities such as mining, refining, and wastewater treatment (e.g. acid mine drainage), copper is a key pollutant. For example, in electrolytic nickel refining, selective removal of copper from nickel electrolysis anolytes has long been a problem plaguing the global metallurgical industry, and requires more effective solutions.^10^**

**Challenges inherent to heavy metal ion remediation from water include incomplete ion removal, high operational costs, and the generation of toxic, indistinguishable ion mixtures in sludge/solid waste streams.^11^ Indeed, isolating and disposing of only toxic contaminants from a myriad of species coexisting in water streams—some of which are valuable catalyst materials—appears to be a costly bottleneck for the water industry. Several physical, chemical, and biological processes have been used to remove heavy metal ions from wastewater, including evaporation, chemical precipitation, coagulation-flocculation, photocatalysis, and membrane or ion-exchange separation, each with advantages and disadvantages.^11^,^12^ Adsorption has been shown to be an effective and economic approach for heavy metal ion removal, as it**

**offers flexibility in design and operation, a low volume of harmful secondary products, reversibility, and is capable of producing high-quality effluent with low energy and maintenance costs.^11^ The efficacy of adsorption methods is limited largely by the ability to design an adsorbent with the necessary physical or chemical functionality for the contaminant of choice.**”

2. On line 84, the authors mention “pH ranges of interest” for copper removal applications. There should be more discussion about these pH ranges of interest and the specific copper removal applications that correspond to specific pH.

Thank the Reviewer for the thorough comment. The statement was edited to point specifically to the pH range where most of current copper adsorbents fail to perform, which is under acidic environments. We have updated this in the main manuscript below.

In the main text (annotated version): page 3, lines 107-111:

“**In general, however, most materials investigated to date for copper ion capture often suffer from one or more drawbacks, such as low selectivity, low capacity, or limited function (typically only in low acidic conditions (pH > 3)), and their syntheses have not been designed for affordable copper removal at scale, often using costly organic solvents or expensive preparations.**”

3. In Figure 1, the authors should also include ChemDraw images of the linkers since it’s hard to view them in the crystallographic pictures.

It is indeed not easy to view the bonding structure clearly from the crystallographic pictures. Thank the Reviewer for pointing this out. We added new Fig. S1 showing the ChemDraw structure of a trinuclear unit of ZIOS into the supporting information, as shown below.

In the Supporting Information:

“

**Figure S1.** Additional view of the structure of a trinuclear unit in ZIOS (A) in ball-and-stick model; and (B) sketched with ChemDraw.

4. The authors suggest that ZIOS is not porous, but from the experimental section, it seems only one activation condition was attempted (100C for 20 hours). Did the authors try any other conditions to activate ZIOS? For a newly reported material, more than one attempt at activation should be performed before suggesting the material is not porous. Alternatively, the theoretical surface area should be calculated using GCMC simulations and compared to the experimental value obtained.

We thank the Reviewer for the excellent point. In response, we have performed additional experiments and calculations (new Fig. S2). We agree that to be able to solidly conclude the non-porous nature of ZIOS, measuring the BET surface area of ZIOS at other activation conditions is essential. In response, we measured the BET surface area of ZIOS after activating the material at 200 ^o^C, which is twice as high that of the original reported condition (i.e. 100 ^o^C). Interestingly, we arrived an almost exact value (e.g. 13.95 m^2^/g) of BET surface area measured for ZIOS at 100 ^o^C (e.g. 13.96 m^2^/g). The fact that more aggressive heating conditions did not influence the measured BET surface areas is consistent with our observation that ZIOS is non-porous.

Furthermore, as suggested by the Reviewer, we theoretically calculated the accessible surface area of ZIOS. Walton and Snurr (JACS, 2007, 129, 8552-8556) proposed and proved that the BET surface areas calculated from the simulated adsorption isotherm (with grand canonical Monte Carlo – GCMC BET) agree very well with the accessible surface areas calculated directly from the crystal structures in a geometric fashion. Specifically, the accessible surface areas were calculated from a simple numerical integration technique where a probe molecule is “rolled” over the framework surface. The fraction of the probe molecules that did not overlap with other framework atoms was then used to calculate the accessible surface area (J. Phys. Chem. C., 2007, 111, 15350-15356). The diameters of the framework atoms for ZIOS were taken from the value of Lennard-Jones parameter, σ, which were used for our MD simulations. As a reference, the value of ZIF-8 was also calculated with the forcefield from (J. Phys. Chem. C, 2011, 116, 933-938). All the accessible surface area below was calculated with a probe diameter of 3.681 Å for nitrogen. The snapshots of framework for the coordination information were collected from the MD simulation.

The accessible surface area of:

ZIOS is 12.36 ±1.66 m^2^/g (20 snapshots’ average)

ZIOS after expansion and contraction is 11.07 ±1.66 m^2^/g (20 snapshots’ average)

ZIF-8 is 1193 ±7 m^2^/g (50 snapshots’ average)

As shown, the values achieved from our theoretical calculation closely track the experimentally measured surface area (e.g. 13.95 m^2^/g for ZIOS and for 1492 m^2^/g ZIF-8). In sum, these results confirm the non­porous properties of our newly designed material – ZIOS. We added the new results and detailed discussion in the text and supporting information, as shown below. Again, we thank the Reviewer for this great comment.

In the main text (annotated version): page 4, lines 146-150:

“The ZIOS structure is essentially non-porous, in contrast to MOFs and ZIFs,^26^ with a Brunauer–Emmett– Teller (BET) surface area of ~12-14 m^2^/g confirmed by both BET measurements at different activation

conditions, e.g. 100 ^o^C and 200 ^o^C and grand canonical Monte Carlo simulations (Fig. 2C and Fig. S2; c.f. BET surface area ~1200 - 1492 m^2^/g for ZIF-8 determined in this work, see the Supporting Information).”

In the Supporting Information:

| “ |  |
| --- | --- |

**Figure S2.** N2 adsorption and desorption isotherms and BET specific surface area of ZIOS after activated at 200 ^o^C for 20 hours. The BET surface area of ZIOS measured at this activation condition (200 ^o^C) replicates almost exactly that of ZIOS measured after activating the sample at 100 ^o^C.”

“*N2 adsorption isotherms and surface area measurements*

N2 adsorption data were collected on an automated three station Tristar 3020 analyzer, connected to a regulated high pressure N2 tank. Powder samples were degassed in vacuum at 100 ^o^C for 20 h before data collection. To validate the non-porous property of the newly reported material like ZIOS, we also measured the BET surface area of ZIOS at another activation condition (e.g., 200 ^o^C for 20 h). At this condition, interestingly, we obtained a surface area value that replicates almost exactly that measured for ZIOS after being activated at 100 ^o^C (e.g. ~13.95 m^2^/g). This result reinforced the non-porous property of ZIOS.

Furthermore, we theoretically calculated the accessible surface area of ZIOS. Walton and Snurr^1^ proposed and proved that the BET surface areas calculated from the simulated adsorption isotherm (with grand canonical Monte Carlo – GCMC BET) agree very well with the accessible surface areas calculated directly from the crystal structures in a geometric fashion. Specifically, the accessible surface areas were calculated from a simple numerical integration technique where a probe molecule is “rolled” over the framework surface. The fraction of the probe molecules that did not overlap with other framework atoms was then used to calculate the accessible surface area (J. Phys. Chem. C., 2007, 111, 15350-15356). The diameters of the framework atoms for ZIOS were taken from the value of Lennard-Jones parameter, σ, which were used for our MD simulations. As a reference, the value of ZIF-8 was also calculated with the forcefield from (J. Phys. Chem. C, 2011, 116, 933-938). All the accessible surface area below was calculated

with a probe diameter of 3.681 Å for nitrogen. The snapshots of framework for the coordination information were collected from the MD simulation.

The accessible surface area of:

ZIOS is 12.36 ±1.66 m^2^/g (20 snapshots’ average)

ZIOS after expansion and contraction is 11.07 ±1.66 m^2^/g (20 snapshots’ average)

ZIF-8 is 1193 ±7 m^2^/g (50 snapshots’ average)

As shown, the values achieved from our theoretical calculation closely represent the experimentally measured surface area (e.g. 13.95 m^2^/g for ZIOS and for 1492 m^2^/g ZIF-8). In sum, these results confirm the non-porous properties of our newly designed material – ZIOS.”

5. Is there a reason for choosing 895ppm (or 425ppm Cu2+) concentration as a starting point? If so, that should be mentioned.

We used 895 ppm (or 425 ppm Cu^2+^) concentration to benchmark the adsorption capability of ZIF-8 reported by Zhang and Wu in (Dalton Trans, 2016, 45, 12653), the work that we selected as a point of reference. We thank the Reviewer for pointing this out. We briefly mentioned this reason in the text, as in:

In the main text (annotated version): pages 4-5, lines 165-169:

“Samples of ZIOS and ZIF-8 were initially exposed to an aqueous CuCl2 solution (895 ppm, i.e. 425 ppm Cu^2+^, selected to benchmark the adsorptive performance of our adsorbents against ZIF-8 reported by Zhang et al.^16^) at room temperature, and within the first 30 min both samples underwent a color change, from white to pale blue or green, respectively (Fig. 2A).”

6. On line 249 (and later), the authors suggest the possibility of transmetalation. ICP-OES should be performed to determine if Zn is present (and at what concentrations) in the water samples being treated. The later ICP data (line 334-335) doesn’t really give this information since the theoretical wt% of Zn in the samples before and after adsorption of Cu is not given.

We thank the Reviewer for this suggestion. In our revised supporting information, we added new Fig. S10 showing the amount of Zn present in the water samples being treated in mg/L. Specifically, we tracked the amount of Zn released according to the initial concentration of copper ion in aqueous solutions. Below is the Fig. S10.

In the Supporting Information:

**Figure S10.** The amount of zinc ion released from ZIOS into the water environment measured in the copper adsorption isotherm tests. The initial concentration of copper ion in aqueous solutions ranges from 0.45 to 2.3, 4.6, 11.5, 23, 46, 103, 207.5 and 415 ppm. The ion concentration was measured with ICP-OES.

**Concentration of zinc ion _(mg/L)_ released from ZIOS into water**

40

70

60

50

30

20

10

0

0	100	200	300	400	500

**Initial concentration of copper ion in aqueous solutions**

**(mg/L)**

We also edited our text accordingly, specifically on page 10, lines 390-394:

“This property is regulated by metal coordination or redox reactions leading to site-selective transmetalation and ultimately to the construction of new heteropolynuclear supramolecular structures. The transmetalation is evident by the amount of zinc ion transferred from ZIOS into the supernatant during copper adsorption quantified by ICP-OES (Fig. S10).”

7. On lines 296-299, the authors discuss how their results contradict previous reports on Cu adsorption and metal-exchange. Are the studies performed in this manuscript done at the same concentrations as those performed previously? The concentrations (Cu:MOF ratio) in this study vs. representative previous studies should be included.

We thank the Reviewer for suggesting such a great point. Specifically, Zhang and coworkers reported a copper adsorption capacity of ~800 mg/g for ZIF-8 after equilibrating 10 mg of ZIF-8 in 30 mL 420 ppm Cu^2+^ solution for 30 min. Meanwhile, we recorded a stable value of adsorption capacity of ~117 mg/g after equilibrating 25 mg of ZIF-8 in 10 mL of 423 ppm Cu^2+^ solution for 24 hours. Based on our calculation, the ratios of [Cu : ZIF-8] used in Zhang’s study and our study are approximately 1.26:1 and 0.17:1, respectively. We included the data on the ratio of mass concentration of Cu : ZIF-8 used in our study and Zhang and coworkers’ study in the manuscript, as shown below.

In the main text (annotated version): page 5, lines 202-206:

“As a comparison at this 30 min mark, the uptake of our sample of ZIF-8 was 151.7 ± 4.3 mg/g. It is worth to note that the ratio of mass concentration (in mg/L) of Cu : ZIF-8 used in our study is ~ 0.17:1, whereas

**that reported in Zhang and coworkers’ work is 1.26:1. We also note that adsorption capacity can be sensitive to slight differences in material preparation and activation conditions.”**

**Reviewer #2 (Remarks to the Author):**

The manuscript from Bui et al. describes their investigation of a novel material for the selective capture of copper ions from water. This material is a framework structure comprised of hydrogen-bonded supramolecular complexes of zinc, 2-methylimidazole and salicylaldoxime (ZIOS). In this submission, the authors study the copper uptake of ZIOS and compare it the metal-organic framework ZIF-8, which has a similar framework composition but lacks the salicylaldoxime functionality. ZIOS, unlike ZIF-8, displayed excellent copper uptake, kinetics and meaningful selectivity thus highlighting this material as a state-of-the-art adsorbent for copper in water. Subsequently, to provide insight to the mechanism responsible for this impressive uptake, adsorption X-ray photoelectron spectroscopy (XPS), synchrotron radiation near-edge X-ray absorption near-edge structure (NEXAFS) spectroscopy and extended X-ray absorption fine structure (EXAFS) spectroscopy were employed to characterise the materials before and after copper adsorption.

This comprehensive experimental analysis identified the salicylaldoxime and imidazole ligands, in ZIOS, are involved in copper coordination, and there is some degree or chemisorption, while ZIF-8 shows the complete transformation of environments, in agreement with the powder X-ray diffraction pattern. Finally, classical molecular simulations were performed to understand the impressive uptake kinetics. The simulations demonstrate a reversible increase in the lattice constant and step-wise diffusion of water, which is considered responsible for the fast adsorption of zinc in this formally non-porous structure.

I believe the quality and potential influence of this work is commensurate with the quality expected by readers of Nature Communications and is publishable subject to revision, following the comments raised below.

We appreciate the Reviewer’s positive perspective on our work, as well as the thorough review. We find that the Reviewer’s comments were quite insightful, which have helped us to revise and strengthen our work. For that, we thank the Reviewer for their time and effort. We address each of the Reviewer’s comments as below.

1. This article is mostly well written and very enjoyable read, however, the description of X-ray spectroscopy characterisation was overly complex and ultimately I am unsure what of value can be inferred from this. I would suggest significantly shortening this section and highlighting concisely what insight this analysis provides.

We thank the Reviewer for this comment. Indeed, we developed this discussion in detail as we are reporting a newly synthesized structure and its behavior. More importantly, as we could not use single crystal X-ray diffraction to dictate the structure of ZIOS-Cu (after copper adsorption), we tried to analyze our XPS and EXAFs data in as detailed a manner as possible to elucidate the structure of ZIOS-Cu. We appreciate your perspective, and have modified the text to best reach a wide spectrum of readers. In response, we have abbreviated this section as far as possible, while moving other parts of the prior X-ray characterization to the Supporting Information. We revised the section as follows below:

In the main text (annotated version): pages 7-8, lines 254-306:

“**XPS analysis of ZIOS-Cu and ZIF-8-Cu was carried out in the binding energy regions of the Cu 2p, Zn 2p, N 1s, C 1s and O 1s orbitals. In-depth analysis is shown in the Supporting Information. We note that for** both materials, we observe signatures of copper(I) in addition to copper(II) (see below). All peak positions were referenced to the C 1s peak of adventitious carbon at 284.5 eV. Initial deconvolution of the Cu 2p spectra for both materials (Fig. 4) revealed similarities with spectra of a previously reported mixed-valence Cu^+^Cu^2+^ complex (for ZIF-8-Cu) and a Cu^+^ complex and/or a heteronuclear Cu^2+^Zn^2+^ complex (for ZIOS-Cu), both supported by macrocyclic ligands.^34^ For ZIF-8-Cu, pairs of Cu 2p1/2,3/2 doublets (at 952.4 / 932.6 eV and 954.8 / 934.8 eV) and the charge transfer shake-up satellites (at 962.8 and 941.8–944.8 eV) were observed, indicative of Cu^+^ and Cu^2+^, respectively. For ZIOS-Cu, the Cu 2p spectrum exhibits peaks at 952.78 and 932.98 eV characteristic of Cu^+^ (2p1/2 and 2p3/2, respectively), in addition to weaker features at 954.48 and 934.78 eV that can be ascribed to Cu^2+^ (2p1/2 and 2p3/2, respectively). The copper(I) peaks could indicate the presence of a five-coordinate Cu^+^ diamagnetic adduct or a four-coordinate Cu^+^ complex with nitrogen macrocyclic ligands, while the copper(II) peaks are indicative of a heteronuclear Cu^2+^Zn^2+^ complex.^36^

In ZIOS, it is possible that copper chelates with salicylaldoxime by partially transmetalating with zinc nodes originally present in ZIOS and/or with 2-methylimidazole by coordinating with the free pyrrolic nitrogen. As seen in Figs. 4 and S8, chelation of salicylaldoxime to Zn^2+^ in ZIOS results in a downshifting of the O 1s binding energies of free salicylaldoxime from 531.68 and 533.08 eV to 531.28 and 532.48 eV (H**O**–N and H**O**–C (phenol), respectively). Similar values of 531.28 and 532.38 eV (H**O**–N and H**O**–C (phenol), respectively) are observed following copper adsorption in ZIOS. In general, coordination to a metal ion results in a shifting of binding energies to lower values as a result of the electron donating/accepting ability of the metal ions.^37^ For ZIOS, the 0.1 eV-downshift of the binding energy of the phenolic oxygen O 1s peak, along with the enhanced intensity of this peak after copper adsorption, show that this site plays an active role in scavenging copper ions. Moreover, the N 1s binding energy of ZIOS exhibits a downshift from 398.88 to 398.78 eV at the pyridinic nitrogen following copper adsorption and, interestingly, a significant increase in intensity of the peak at ~400.18 eV for C=**N**(–O) and/or a pyrrolic nitrogen (Figs. 4 and S8). Often, fluctuations of peak intensity at a given chemical state are related to changes in the number of atoms in that state. The remarkable increase of the intensity ratio of I(C=N)/I(C–N) from ~0.65 for ZIOS to ~1.54 for ZIOS-Cu indicates significant translational/rotational movement of C=N(–O) and/or pyrrolic nitrogen bonding from the bulk to the surface sites at which copper ions are coordinated. It remains unclear what imidazole nitrogen participates in copper coordination. Given the tautomeric property of the ionizable imidazole ring, both are possible if the Zn-imidazole bond was cleaved at the pyridinic nitrogen by copper ions. Note that the pH value of the stock copper chloride solution is ~ 5.5.”

2. The transformations of ZIF-8 in an aqueous copper environment are not overly surprising given the propensity for biomolecule@ZIF-8 phase changes in response to post-synthesis treatments (e.g. washing) [10.1021/acs.chemmater.7b04977, 10.1039/c8mh01611a]. The mechanism present in these studies is also likely to be present here, and responsible for the crystal-to-crystal transformation observed.

We thank the Reviewer for bringing these works to our attention. We included the reference accordingly, as shown below:

In the main text (annotated version): page 5, lines 177-181:

“In contrast, after 24 hours the ZIF-8 particles decrease in size and in general take on a different morphology (Fig. 2A). Crystal-to-crystal transformation due to post-synthetic treatments were previously

reported for ZIF-8.^27^ Powder X-ray diffraction characterization revealed that both materials undergo a transition to a new crystalline state upon Cu^2+^ uptake (Fig. 2C).”

with reference 27 to be:

“27. Liang W, Ricco R, Maddigan NK, et al. Control of Structure Topology and Spatial Distribution of

Biomacromolecules in Protein@ZIF-8 Biocomposites. *Chem Mater*. 2018;30(3):1069-1077. doi:10.1021/acs.chemmater.7b04977”

3. As the crystal morphology is not overly changed by the process of Cu adsorption, and good crystallinity is present, were single-crystal X-ray diffraction experiments performed? As this would unequivocally show the location of Cu.

We thank the Reviewer for this interesting point. Yes, we had the same thought and we did indeed try to conduct the single-crystal X-ray diffraction experiments of this Cu-adsorbed materials several times. However, we were not able to achieve a crystal structure as after copper adsorption. After discussing with the beamline scientists, one possible reason is that the ZIOS-Cu material was most likely present in its 2D form after Cu uptake, as evidenced by SEM images that we did not show in our original manuscript. Thanks to the Reviewer’s question, we now include those images (new Fig. S3), as well as a brief discussion on our efforts on acquiring the crystal structure of Cu-adsorbed material. Also, the fact that the material is presenting in their 2D form is very interesting, and perhaps will open a new path for research in this area.

In the Supporting Information:

**Figure S3.** Representative scanning electron microscope images of the two-dimensional sheet-like structure of some domains of ZIOS-Cu.

In the main text (annotated version): page 5, lines 173-177:

“In other domains of ZIOS-Cu, we observed a two-dimensional sheet-like structure (Fig. S3) which is likely due to the sequence of sonication, vortex, copper adsorption, centrifugation, washing, and drying that the material underwent. The single crystal structure of ZIOS-Cu was thus not obtained due to the lack of sufficiently large crystals that satisfy the minimal size requirement of the single crystal X-ray diffraction technique.”

4. It is stated that transmetalation in ZIOS does occur, evidenced by the leaching of Zn after adsorption. How problematic is this for the application of the material? Moreover, was the selectivity of Cu in a mixture containing Zn investigated?

We thank the Reviewer for the excellent point, however, we believe this is not a major issue which we explain with our data and analysis below (new Fig. S10). From our perspective, although the partial leaching of Zn after adsorption is not ideal, when exploiting ZIOS for applications such as acid mine drainage-

polluted water treatment, or Cu/Ni separation in the refining of nickel, the treatment (if necessary) of a stream having ~ 60 ppm of Zn^2+^ leached out of ZIOS (after being used to selectively reduce Cu^2+^ concentration from ~425 ppm to ~1.5 ppm) is readily obtainable. Furthermore, obtaining a deterministically designed material that can selectively perform at low concentration range, in acidic environment, and in complex mixtures of ions is not a small feat.

In our revised supporting information, we added Fig. S10 showing the amount of Zn present in the water samples being treated in mg/L. Specifically, we tracked the amount of Zn released according to the initial concentration of copper ion in aqueous solutions.

In addition, thanks to the Reviewer’s suggestion, we evaluated the selectivity of ZIOS toward copper in a mixture simultaneously having cations Na^+^, Ca^2+^, Fe^3+^, Ni^2+^, and Cu^2+^, and in the presence of Zn^2+^. Results are tabulated in the new Table S6, as also shown below.

In the Supporting Information:

**Figure S10.** The amount of zinc ion released from ZIOS into the water environment measured in the copper adsorption isotherm tests. The initial concentration of copper ion in aqueous solutions ranges from 0.45 to 2.3, 4.6, 11.5, 23, 46, 103, 207.5 and 415 ppm. The ion concentration was measured with ICP-OES.

**Concentration of zinc ion**

**released from _(mg/L)_ ZIOS into water**

40

70

60

50

30

20

10

0

0	100	200	300	400	500

**Initial concentration of copper ion in aqueous solutions**

**(mg/L)**

We also edited our text accordingly, specifically on page 10, lines 390-394:

“This property is regulated by metal coordination or redox reactions leading to site-selective transmetalation and ultimately to the construction of new heteropolynuclear supramolecular structures. The transmetalation is evident by the amount of zinc ion transferred from ZIOS into the supernatant during copper adsorption quantified by ICP-OES (Fig. S10).”

In the Supporting Information:

“**Table S6.** Copper selectivity of ZIOS when exposed to aqueous solutions simultaneously containing equimolar concentration (~ 10 ppm) of Na^t^, Ca^2t^, Fe^3t^, Ni^2t^, Cu^2t^, and ~30 ppm of Zn^2t^.

| An aqueous solution having 10 ppm of Na^t^, Ca^2t^, Fe^3t^, Ni^2t^, and Cu^2t^, co-existing with 30 ppm Zn^2t^ | | | | |
| --- | --- | --- | --- | --- |
| Before adsorption |  | Cu^2t^ | Ni^2t^ | Zn^2t^ |
|  |  | 9.5 ± 1.2 | 12.4 ± 0.7 | 32.1 ± 1.9 |

**After 24-hr adsorption	as-prepared	0*	0*	21.8 ± 0.3**

| **pH was adjusted to 2.48** | **1.2 ± 0.2 13.5 ± 0.02 48.9 ± 2.3** |
| --- | --- |

***undetectable**

**The initial concentration ratio of Cu^2+^: Zn^2+^ is 1:3. As Ni^2+^, Cu^2+^, and Zn^2+^ are the three ions of interest (based on the previous selectivity test without the presence of Zn^2+^), we tracked the concentration of these three cations before and after adsorption with ICP-OES. Below is the table showing the concentration of these ions before and after adsorption. Results show that the copper selectivity of ZIOS in an aqueous environment having Zn^2+^ at three times more concentrated (e.g. Cu^2+^: Zn^2+^ = 1:3) remained unchanged. Interestingly, at pH 2.48, the Cu/Ni selectivity behavior of ZIOS is again observed. ZIOS appears to not uptake Ni^2+^ at this pH value. Meanwhile, at as-prepared pH, ZIOS appears to have some capacity to further uptake Zn^2+^ presenting in the solution on top of Cu^2+^ and Ni^2+^.”**

In the main text (annotated version): page 6, lines 234-243:

**“Indeed, when adsorption tests were carried out using metal ion solutions prepared with pH = 2.45, copper uptake by ZIOS remained high (~97.5%) while the material adsorbed negligible iron (~14.3%) and nickel (~2.9%); in contrast, ZIF-8 adsorbed more iron (~30.0%) than copper (~14.6%) (Fig. 3E). Significantly, these results for ZIOS at lower pH may be promising for applications such as electrolytic nickel refining, where the separation of copper and nickel ions remains a substantial challenge.^10^ For example, the Cu^2+^/Ni^2+^ separation factor achieved with ZIOS at pH 2.45 is 1281.1, while separation factors of 1.2 (pH = ~5) and 4.1 (pH = 4) were reported for the commercial resin Purolite S984^28^ and an *N*-octyl-2-aminomethylpyridine-functionalized chelating resin^29^ under equimolar concentrations of Ni^2+^ and Cu^2+^. We further evaluated the copper selectivity of ZIOS in an ion mixture having Zn^2+^ as one of the contaminants and found this behavior almost unchanged (Table S6).”**

5. The breathing effect demonstrated by molecular simulation, while interesting, is hardly comparable to the experimental system. Firstly, the molecular simulations show behaviour reminiscent of the swelling of clay structures [10.1063/1.1493186]. Although it is stated that the hopping of water molecules goes from hydrogen bonding sites there is no evidence of this reported. It could just as likely be sequential hydration of interlayer sites, as the material is indeed a two-dimensional framework. The radial distribution functions (RDF) display does not provide sufficient evidence for H-bonding sites. For example, the hydrogen atoms of the benzene group are unlikely to be involved in hydrogen bonding. The methodology behind the calculation of the hopping rate is not presented and I would be hesitant to compare this directly to Aquaporin-1. Furthermore, this swelling mechanism (I would refrain from using the term breathing as this is often associated with a non-monotonic volume change upon adsorption) is not observed experimentally, despite the comprehensive experimental characterisation, why?

As this comment has different points, we would like to address each of these points in detail as follows below:

(a) The breathing effect demonstrated by molecular simulation, while interesting, is hardly comparable to the experimental system.

We agree that it is quite challenging to provide direct in-operando evidence to support our hypothesis of the breathing effect at this stage as it would require advanced characterization tools still under development

at synchrotron beamlines. However, we believe we do have an empirical basis for this claim supported by our powder XRD data on top of the expanding/contracting behavior induced from the reversible lattice constants calculated by molecular simulation, although this may be interpreted or discussed in different ways. Specifically, with XRD, we proved that the crystalline structure of the as-synthesized ZIOS stay the same after being vigorously treated by soaking in a water environment for 2 months, washing thoroughly with methanol, sonicating, centrifuging, and drying in vacuum. Undergoing such a harsh process, yet ZIOS still maintains its basic crystalline structure with an almost exactly the same XRD pattern. To an extent, this data confirms the structural integrity of our crystal, which is built upon a network of H-bonding, even after going through cycles of washing/swelling-drying, or in other words expanding - contracting. The fact that ZIOS crystalline structure comes back to its original structure after being excessively exposed to water agrees very well with our MD simulations showing that after removing water from the cell structure, all lattice constants (in three directions Lx, Ly, and Lz) return back to their original values. We thus believe that this reversible behavior can be called a breathing effect. We can also rephrase this if it is confusing terminology.

We do agree that more (in-operando) experiments should be done in the future to prove this truly interesting behavior, but we do note that this is not a central claim of the manuscript so we can moderate this hypothesis if needed. We however hope that our hypothesis and partial experimental evidence can inspire scientists in the field to further explore and, better yet, leverage this behavior for various applications. Furthermore, to an extent, this behavior is analogous to what was reported for some metal organic frameworks which act as hosts that can adapt their pore opening to accommodate guest species, thereby producing a dramatic increase or decrease in cell volume without a loss of crystallinity or bond breaking. In our cases, the new material does not just behave instantaneously. Instead, it held its structure after being immersed in water environment for 2 months, and then recovered its structure after drying up. We thank the Reviewer for the comment from which we realize that we did not provide enough clarification on these data through thorough discussion. We thus revised the corresponding paragraph as below.

In the main text (annotated version): page 10, lines 398-419:

**“Our results show that immersion of ZIOS unit cells in water leads to an expansion of the crystal lattice and the formation of nanochannels that can accommodate water molecules through the propagation of a hydrogen bonding network. Water incorporation occurs quite rapidly**—**a snapshot at 15 ns (Fig. 6A) shows a noticeable amount of water molecules intercalating into ZIOS channels. Furthermore, as the number of water molecules introduced into the ZIOS unit cell increases, a gradual lattice expansion occurs in all crystallographic directions** – **Lx, Ly, and Lz (Fig. 6B,C). When water is removed, the lattice contracts back to its original size within less than 1 ns (Fig. 6B,C), indicating the expansion is reversible. Indeed, powder X-ray diffraction characterization of a sample of ZIOS soaked in water for 52 days, washed with methanol, and dried in vacuum revealed an almost identical pattern to that simulated from single crystal X-ray diffraction data (Fig. 6D). A slight shift of the peaks is observed due to different diffraction scans temperatures (room temperature for the PXRD data *versus* 100 K for the synchrotron single crystal X-ray diffraction data). This result serves as an experimental evidence of the breathing phenomena, suggested by the MD simulation, where ZIOS recovers its original crystalline structure after being excessively exposed to water for ~ 2 months. Other experimental approaches are however necessary to observe this truly interesting behavior in-operando. Still, to an extent, the breathing behavior of ZIOS is analogous to what was reported for some metal-organic frameworks that can adapt their pore opening to accommodate guest species, leading to dramatic change in cell volume without a loss of crystallinity or bond breaking.^55,56^ In our cases, the new material does not just behave instantaneously. Instead, it held**

**its structure through vigorous treatment steps. The impressive structural integrity of ZIOS supports the presence of a strikingly robust hydrogen bonded network which helps the material to reversibly breathe through expansion–contraction cycles before and after exposure to water.**”

(b) Firstly, the molecular simulations show behaviour reminiscent of the swelling of clay structures [10.1063/1.1493186].

Indeed, the work that is recommended by the Reviewer, to some extent, shares a somewhat similar approach in understanding the microscopic behavior of the sheet-built 3D crystalline material. We, however, believe that the mechanism of swelling of these clays (hereinafter referred to the clay structure studied in [10.1063/1.1493186]) are not the same as that of ZIOS due to the following reasons below. First, our crystal is shown by the single-crystal XRD data to be built on a network of strong H-bonding active sites, whereas the clay structures have fewer H-bonding sites. Second, unlike ZIOS, charge density plays a critical role in the non-monotonic swelling mechanism of these clays. Our model also took one step further in confirming the reversible change of the lattice constants in three directions (L_x_, Ly, and L_z_) between adding and removing water molecules. Third, the interlayer distance between the sheets in these clays are significant, often > 10 Å. The interlayer distance in our material is the average length of an H-bonding which is 2.7 Å, still smaller than the van der Waals (vdW) diameter of a water molecule. The mechanism of water interpenetrating into these clays and ZIOS is thus believed to be different. We further elaborate these points as below.

There are three main mechanistic differences between these clays (C0.75[Si8](Al3.25Mg0.75)O20(OH)4, the clay structures studied in [10.1063/1.1493186]) and ZIOS:

1. Charge density plays a critical role in the swelling mechanism of these clays. In clay structure, there
   exists a much more mobile multivalent cation network, which leads to a significant cation displacement and the presence of a network of counterions between the two layers that are responsible for a dynamic and non-monotonic movement of water, i.e. the crystalline swelling in water. In C0.75[Si8](Al3.25Mg0.75)O20(OH)4, the only substitutions are octahedral while all of the negative charges of the clay are localized in the middle of the clay sheets (i.e. away from the surfaces). Swelling occurs through the specific interactions between the metal and the surfaces or solvation of the metal center. Unlike clays, we have Zn^2+^ as the only metal center in ZIOS. The Zn^2+^ sites are not available or exposing onto the surface of the sheet, but rather busy coordinating to its surrounding ligands. Therefore, these Zn^2+^ centers stay almost fixed in the pure water environment.
2. The interlayer distance in these clays is approximately 10 – 12 Å. In ZIOS, the interlayer distance is determined by the H-bonding length, which is experimentally measured to be 2.7 Å by single-crystal X-ray diffraction (SCXRD). Meanwhile, the vdW radius of water is ~3.166 Å. In fact, SCXRD does show evidence for a prosperous network of H-bonding. This experimental result suggests that water molecules first need to interact with the crystal structure through H-bonding with the host, before gradually opening up the channels to a degree that more molecules can come in and start building layers of molecules in between these layers. Furthermore, given the small interlayer distance between sheets in ZIOS, i.e. 2.7 Å, if water formed within the layer through solvation only, oftentimes the water structure would be more ice-like, unless the water interact with the host ZIOS through its rich H-bonding network.
3. Regarding the simulation methods, analogous Monte Carlo and MD simulations were used in [10.1063/1.1493186]. Our MD simulation approach, however, was conducted with some

significant improvements. In [10.1063/1.1493186], the lattice expansion was evaluated by Monte Carlo which cannot deal with the dynamics of water molecules. Instead, the water molecule dynamics was tested at the frozen ensemble condition (NVE) with the rigid structure model, namely, sheets are frozen in a stable configuration (obtained beforehand by Monte Carlo) and considered as rigid molecules while water molecules were only allowed to move around in a two-dimensional domain. On the other hand, we used a flexible structure with CADA model for zinc atoms. The ZIOS structure and water molecules exchange their potential field momentarily to practically describe the movements of water molecules and the expansion-recovery of ZIOS structure. We believe that it is imperative to present the water molecules’ 3D directional movement between stable sites as shown in Fig. 6E. This approach allows us to simulate the reversible change of lattice constant between adding and removing water molecules into / out of the unit cell (expansion-contraction) which was not discussed at all in [10.1063/1.1493186].

(c) Although it is stated that the hopping of water molecules goes from hydrogen bonding sites there is no evidence of this reported. It could just as likely be sequential hydration of interlayer sites, as the material is indeed a two-dimensional framework.

Yes, it could just as likely be sequential hydration of interlayer sites. However, unlike the clay structure where the interlayer distance is ~12 Å, which is several times that of the van der Waals diameter of a water molecule, the interlayer distance in our material is smaller than the vdW diameter of a water molecule, that means that sequential hydration of interlayer sites is not occurring readily after the interlayer distance is gradually opened up by individual water molecule interacting with H-bonding active sites through hopping. In our MD simulation, we also discussed the transition from hopping to diffusion.

More specifically, we present support for the hopping of water molecules in Fig. 6E. If the water molecules move sequentially on the hydration interlayer sites, the frequency would valley at 7-9 Å on the x-axis range. The main H-bonding acceptor site is the nitrogen atom in imidazole (N2), as shown in Fig 6F and Fig. S13. The first peak of RDF between Hw-N2 has the highest intensity (Fig. 6E). Interestingly, the intensity of this peak sequentially increases with the number of water molecules (Fig. S13A). For the other main stable sites such as OH and HA (Fig. S13C-D), the RDF peak intensity does not dramatically change with the number of water molecules, which means that, unlike N2, these sites restrictively attract water molecules. Furthermore, the peak radius around 2 Å confirms the strong attractive interactions between water and these N2 sites, compared to a typical H-bonding distance, e.g. reported in [Phys. Rev.Lett. 85, 5583–5586 (2000)].

(d) The radial distribution functions (RDF) display does not provide sufficient evidence for H-bonding sites. For example, the hydrogen atoms of the benzene group are unlikely to be involved in hydrogen bonding.

We appreciate the Reviewer’s perspective here, and agree to some extent. Our central basis for this claim was the experimental data, we used the simulations to see if they agreed or disagreed with the observed data. Thus, we use RDF analysis as an approach to compare differences between distinct H-bonding sites. It is the single-crystal X-ray diffraction experiment that provides structural evidence of the fact that our new material is built on the propagation of an H-bonding network.

Regarding the suggestion that “the hydrogen atoms of the benzene group are unlikely to be involved in hydrogen bonding”, several studies have shown that the center of a benzene ring typically act as a hydrogen bond acceptor, and can interact significantly with a hydrogen bond donor (like the > NH group). For e.g., Levitt and Perutz (*“Aromatic rings act as hydrogen acceptors”,* J. Mol. Biol., 1988, 201, 751-754) shows that this interaction, which is about half as strong as a normal hydrogen bond, is expected to play a

significant role in molecular associations. Also, the aromatic hydrogen bond arises from partial charges centered on the ring carbon and hydrogen atoms which play a role in forming strong interaction observed in polymer–drug complexes. Benzene was also proven to form Hydrogen bonds with ammonia dimer (*“Hydrogen bonding in the benzene-ammonia dimer”,* Nature, 1993, 362, 735-737), alcohol (*“Hydrogen bonding with aromatic rings”*, AIChE Journal - Thermodynamics, 2001, 47, 948-953), (though weak, is believed by the authors to significantly contribute to the chemical potential of the molecules.), and liquid water. Using Raman scattering, Dor Ben-Amotz and coworkers (*“it-hydrogen bonding in liquid water”*, J. Phys. Chem. Lett., 2011, 2, 2930-2933) show that liquid water can form it-hydrogen bonds with benzene and other aromatic rings (e.g. phenol, phynelalanine), with the bonds forming with nearly every benzene molecule. The authors also showed that although a water-benzene bond is weaker than a water-water hydrogen bond, water molecules associated with benzene have more rotational and translational freedom than their water-water counterparts, the it-hydrogen bond are entropically favored. They also suggested that it-hydrogen bonding may be important for biological binding, recognition, and signaling processes involving aromatic groups (*“Liquid water forms hydrogen bonds with benzene”*, Chemical & Engineering News, 2011, 89, 31). We revised the main text accordingly to briefly mention this behavior, as shown below.

In the main text (annotated version): page 11, lines 434-445:

“**In ZIOS, the most active hydrogen acceptor site was determined to be the pyridinic imidazole nitrogen, based on the radial distribution function, *g*(r) (see Fig. 6F and the Supplementary Information). Meanwhile, the most active hydrogen donor sites are the hydrogen atoms of the hydroxyimino groups. From our simulations, the hydrogen bonding peak was observed within 2 Å for the most active sites (Fig. S13). Similar to previously reported,^57-60^ we found evidence of the contribution of the benzene rings to ZIOS molecular associations through the hydrogen bonding network. Although a water-benzene bond is weaker than a water-water hydrogen bond, water molecules associated with benzene were believed to have more rotational and translational freedom than their water-water counterparts.^59^ Further exploration is essential to confirm this hopping behavior experimentally.** ”

(e) The methodology behind the calculation of the hopping rate is not presented and I would be hesitant to compare this directly to Aquaporin-1. Furthermore, this swelling mechanism (I would refrain from using the term breathing as this is often associated with a non-monotonic volume change upon adsorption) is not observed experimentally, despite the comprehensive experimental characterisation, why?

We thank the Reviewer for this concern, and agree. In this revised manuscript, we deleted the comparison between the hopping rate of water molecules in our structure to transport in Aquaporin-I as this is at the very early stage. We also changed the language to maintain the reservation we have as of now. Regarding the swelling mechanism, as aforementioned, MD shows that lattice constants calculated in x, y, and z directions recover to their original values after water molecules are removed out of the ZIOS cell. Also, our XRD data proved that ZIOS recovers back to its original crystalline structure after immersing the crystal in water for 2 months. This say that the volume change in water environment perhaps is NOT non-monotonic, but indeed expanding-contracting, rather than an uncontrollable swelling process. We would like to note that our calculation was done in pure water environment, not having copper so we won’t say it as an “adsorption”.

**Reviewer comments, second version:**

Reviewer #1 (Remarks to the Author):

The authors completed all revisions thoroughly and carefully. They considered all points raised by the reviewers, and addressed them sufficiently. As such, there are no further recommendations for revisions and I believe the manuscript is suitable to be published in Nature Comms.


Reviewer #2 (Remarks to the Author):

The research article from Bui et al. was revised and my previous comments were carefully considered, and thoroughly addressed.

The revised manuscript presents a high-quality investigation and relevant to a wide audience. I have no further questions or comments and I highly recommend this article for publication in Nature Communications.

**Author rebuttal, second version:**

**Reviewer comments, third version:**

**Author rebuttal, third version:**
